# Reliability, construct validity, and usefulness of an assessment device for agility and motor-cognitive reactive stepping performance in community-dwelling older adults

**DOI:** 10.1371/journal.pone.0351843

**Published:** 2026-07-17

**Authors:** Florian Giesche, Lutz Vogt, David A. Groneberg, Thorben Hülsdünker

**Affiliations:** 1 Institute of Occupational, Social and Environmental Medicine, Goethe University Frankfurt/Main, Frankfurt/Main, Germany; 2 Department of Sports Medicine and Exercise Physiology, Goethe University Frankfurt, Frankfurt am Main, Germany; 3 Department of Sport, LUNEX, Differdange, Luxembourg; 4 Luxembourg Health and Sport Sciences Research Institute (LHSSRI), Differdange, Luxembourg; ASPIRE Academy for Sports Excellence, QATAR

## Abstract

Age-related cognitive and motor decline impairs daily functioning and increases fall risk. Although both domains interact in everyday activities, they are often assessed separately, limiting ecological validity. This study examined reliability and construct validity of an assessment device for agility and motor–cognitive reactive stepping performance in older adults. In a test–retest design, 72 older adults (48 women; 73.7 ± 7.6 years) completed agility tests (Random Star Run and a dual task with multiple object tracking) and motor-cognitive tasks on the SKILLCOURT across four sessions: familiarisation, test day 1 and day 2 (7 days apart), and test day 3 (3 months later). Agility tasks required reactive changes of direction in response to visual cues, while motor-cognitive stepping tasks assessed simple and choice reaction time, task switching, 2-back, and Stroop word-color interference. Intersession Reliability was evaluated using intraclass correlation coefficients (ICC) and coefficients of variation (CV), learning effects with linear mixed models, and construct validity via age-adjusted regression against established motor (Timed-up-and-Go; TUG; single-/dual task) and seated PC-based cognitive measures of reaction speed and executive functions corresponding to the SKILLCORT tasks. Significant learning effects were observed mainly between test days 1 and 2. Relative reliability was moderate to excellent for SKILLCOURT agility, simple reaction time, and Stroop word-color performance (ICC = 0.70–0.94), but lower for choice reaction time, task switching, and the 2-back task (ICC = 0.49–0.66). Absolute reliability was generally good (CV = 4.6–7.2%), except for the SKILLCOURT task switching and 2-back (~12%) and, in particular, the dual task agility test (32%). Random Star Run agility was predicted by TUG single-task performance, PC-based Stroop interference control, and simple reaction time (R^2^ = 0.77), while dual task agility was predicted by TUG dual task and PC-based 2-back performance (R^2^ = 0.37). All motor-cognitive outcomes were significantly associated with corresponding PC-based cognitive measures (R^2^ = 0.20–0.41). The SKILLCOURT demonstrated good reliability for the Random Star Run agility test and most motor-cognitive measures, although adequate familiarisation appears necessary to minimise learning effects. Construct validity was confirmed for agility measures, while reactive stepping tasks appeared to involve greater motor demands and task interference, which may weaken correlations with comparable PC-based cognitive tests.

## Introduction

Aging is associated with a progressive decline in motor performance (e.g., gait, balance, lower limb strength, walking and movement speed) and cognitive performance (e.g., processing speed, memory, and executive functions) [1–4]. Both domains are essential for daily activities, ranging from basic tasks such as feeding and dressing to more complex ones, such as crossing streets or performing dual tasks like walking while talking [5]. Consequently, declines in motor and/or cognitive function can impair daily functioning, reduce quality of life, and increase fall risk [1,6,7].

Motor and cognitive processes are closely linked through shared brain structures and networks. Clinically, this is reflected in the high prevalence of falls among older adults with cognitive impairment or dementia [8,9]. Evidence indicates that lower motor function is related to faster cognitive decline and age-related structural brain changes (e.g., white matter lesions, smaller total brain and cerebellum volume, frontal and primary motor cortex atrophy) [1,10]. Older adults also exhibit greater dual task costs [11], defined as declines in motor (e.g., walking or balancing) or cognitive performance (e.g., recalling digit spans) under dual task compared with single-task conditions [12]. These costs likely reflect increased compensatory demands on limited attentional resources, as older adults recruit additional neural resources to offset sensorimotor declines and reduced automatization of gait and balance through increased cognitive movement control [13–17]. This shift from more automated to increasingly cognitively controlled movement may lead to capacity limitations, leaving insufficient resources to manage motor deficits in cognitively demanding situations, reflecting age-related neural inefficiency [1,18]. As attentional and executive resources become increasingly shared between motor and concurrent cognitive task demands, greater overlap in neural resource recruitment and competition for processing capacity may occur, particularly within higher-order associative brain regions, ultimately resulting in structural (generic) interference [19]. This is particularly relevant because fronto-striatal networks involved in attentional and executive control, including connections between the prefrontal cortex and basal ganglia, also undergo age-related decline, potentially impairing the ability to cope with combined motor and cognitive demands [1].

Despite the interplay between motor and cognitive functions, these domains are typically assessed separately [7,20]. According to the World Guidelines for Falls Prevention [7], multifactorial fall risk assessments include both motor (e.g., Timed Up and Go, Chair Stand. balance, walking speed under single- and dual task conditions) and seated computer- or pen-and-paper-based cognitive measures (e.g., executive functions). However, many daily-life tasks require continuous motor-cognitive integration, in which cognitive processing is a prerequisite for task completion [21], including rapid decision-making and reactive movement responses or adaptations [22]. These demands align with the concept of agility, which encompasses coordinated rapid and reactive whole-body movements in response to visual stimuli [23], such as responding to traffic signals or avoiding vehicles, cyclists, pedestrians, or other obstacles, requiring rapid processing speed, executive functioning, and lower-limb strength [24,25]. Agility is well established for injury prevention [26,27] and team sport performance [24,28], but remains largely unexplored in older adults [29]. Promising motor-cognitive reactive stepping paradigms performed in response to basic visual and executive cognitive stimuli have been associated with cognitive decline and fall risk in older adults [14,30–33].

While such integrated approaches may complement more isolated motor and cognitive assessments by improving ecological validity [34] and supporting the early detection of subtle functional decline and fall risk in community-dwelling older adults, they remain limited in current practice. Existing motor–cognitive stepping paradigms (see above) are heterogeneous across studies with respect to protocols and assessment tools and are typically applied separately, with agility and reactive stepping rarely being assessed within a standardized, integrated framework. The SKILLCOURT technology integrates both agility and reactive stepping tasks with basic visuomotor reaction and executive function demands within a unified assessment environment [35,36]. Although its reliability and construct validity have been demonstrated in athletes [24,35,36], evidence in older adults is lacking.

Therefore, the present study addresses this gap by investigating the intersession reliability, usefulness (sensitivity to detect meaningful performance changes), and convergent construct validity of selected SKILLCOURT agility and motor-cognitive reactive stepping assessments in community-dwelling older adults. We hypothesized good reliability and usefulness, but only moderate associations with standard PC-based cognitive tests due to differences in motor–cognitive integration and physical demands compared with seated cognitive assessments, which may introduce additional motor and cognitive interference.

## Materials and methods

### Design and ethics

To assess the reliability of the selected agility and motor-cognitive SKILLCOURT tests (SKILLCOURT GmbH, Bergrheinfeld, Germany), a test-retest study design was adopted. The present study included four appointments while the first one served as a familiarisation session for the SKILLCOURT tests (no data assessment) to minimise potential learning effects as recommended by a previous study with athletes [35]. All appointments (familiarisation, test day 1 and 2) were scheduled seven days apart, and a retention test (test day 3) was conducted after three months. All measurements were conducted independently across sessions. To assess the construct validity of the SKILLCOURT tests, only data of test day 1 was used.

The study was approved by the local Ethics Committee of the Faculty of Psychology and Sport Science at Goethe University Frankfurt/Main, Germany (reference number: 2023−02) and was registered in the German Clinical Trial Register (ID: DRKS00034524). Although the trial was registered for two study sites (Goethe University Frankfurt/Main, Germany, and LUNEX, Luxembourg), the present analysis includes data from only one site (Goethe University Frankfurt), as no test-retest design was implemented at the second site, precluding reliability analyses. Participants were informed about the study procedures, and written informed consent was obtained prior to participation. All procedures were conducted in accordance with the ethical standards of the Declaration of Helsinki.

### Study procedure

The familiarisation session as well as test day 1 and 2 started with the assessment of participant characteristics (questionnaires). Following the questionnaires, participants completed a 5-minute standardised warmup (comfortable walking, knee and heel raises, side steps, walking lunges, wall push-ups, calf and hip flexor stretching, and trunk and shoulder mobility; ~ 30 sec each) before actual testing. During the first appointment, all SKILLCOURT tests were explained and demonstrated. Participants were given the opportunity to ask questions and completed at least one full familiarisation trial for each task. Additional practice trials were allowed until participants indicated that they understood the task. Familiarization procedures (instruction, demonstration, and practice trials) were standardised across all SKILLCOURT tests.

**Fig 1:**
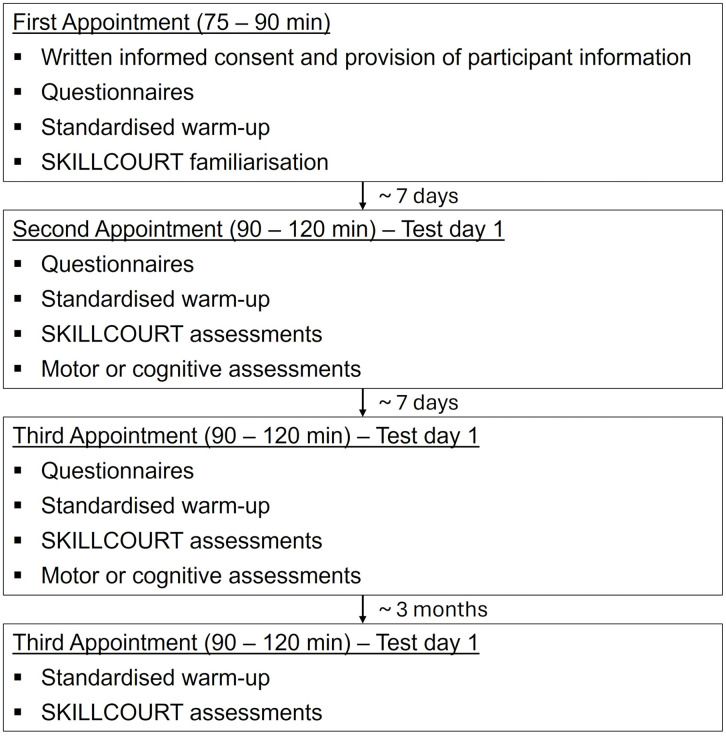
Overview about the study procedure.

The order of SKILLCOURT tests subdivided into the categories of lower extremity reaction time, executive function tests, and agility tests was block-randomised and balanced for each participant prior to the familiarisation session to minimise potential order- and fatigue-related effects. This order was kept constant across all three test days. On test days 1 and 2, participants completed conventional motor assessments on one day and cognitive PC-based assessments on the other day, with the order randomized across participants. On test day 3, only the SKILLCOURT tests were conducted. Participants were instructed to perform all tests as quickly and accurately as possible while prioritising safety. No verbal encouragement was given and no performance feedback was provided. For all tests, the best trial (or the only trial, where applicable) was used for analysis. A schematic overview of the testing protocol is provided in Fig 1.

### Participants

Participants were recruited using convenience sampling through local community advertisements, senior networks, and a local newspaper. Recruitment took place in two phases: between 6 February and 15 May 2023 and between 12 February and 15 March 2024. Data assessments were conducted between 15 May and 1 November 2023 and between 15 March and 1 November 2024. Eighty functionally independent older adults participated in this study. Participants were excluded if they (1) were restricted in independent activities of daily living (e.g., washing, personal hygiene, dressing, shopping, toileting, or continence), (2) required formal care, (3) reported acute or chronic conditions that could impair physical or cognitive performance, including neurodegenerative or other cognitive disorders, thereby limiting test participation, (4) had undergone surgery under general anaesthesia within the previous 6 months or minor surgery without general anaesthesia within the past 3 months, (5) used mind- or perception-altering substances, (6) were unable to walk for at least 10 minutes at a comfortable pace without stopping or using walking aids, (7) experienced acute pain or injury, or (8) had sustained a head injury or concussion within the past 6 months. Participants were asked to avoid strenuous physical activity for 48 hours prior to each test day and to refrain from consuming substances such as alcohol or nicotine.

No a priori sample size calculation was conducted. The sample size was considered sufficient for estimating reliability and validity metrics and for linear regression analyses with a limited number of predefined predictors and exceeds that of comparable validation studies in athletes [24,35,36] to account for the greater variability expected in older adults.

### Assessments

#### Participant characteristics.

The following parameters and questionnaires (German versions) were collected: Age, anthropometrics, formal education (years of schooling and highest graduation), health-related quality life (SF-12; [37]), depressive symptoms (Geriatric Depression 15-Scale; [38]), cognitive function (Montreal Cognitive Assessment, MoCA; [39]), physical activity level (single-item physical activity screening tool; [40]), number of individuals who experienced at least one fall in the previous 12 months (defined as “an unexpected event in which the participants come to rest on the ground, floor, or lower level” [41]), and fear of falling (Short FES-I Form; [42]). Physical function tests included handgrip strength assessed with a SAEHAN hydraulic hand dynamometer (best of three trials per hand), known as an important indicator of overall health, physical capability, muscle function and sarcopenia in older adults [43]. Lower-extremity strength was assessed using the 30-second Sit-to-Stand Test [44] (number of full stands completed from a 17-inch [43.2 cm] chair; best of three trials). Visual acuity was assessed using a standardised Landolt C vision test, confirming sufficient vision (≥ 0.7; [45]). All participants were able to perceive colors.

#### SKILLCOURT tests.

**Fig 2:**
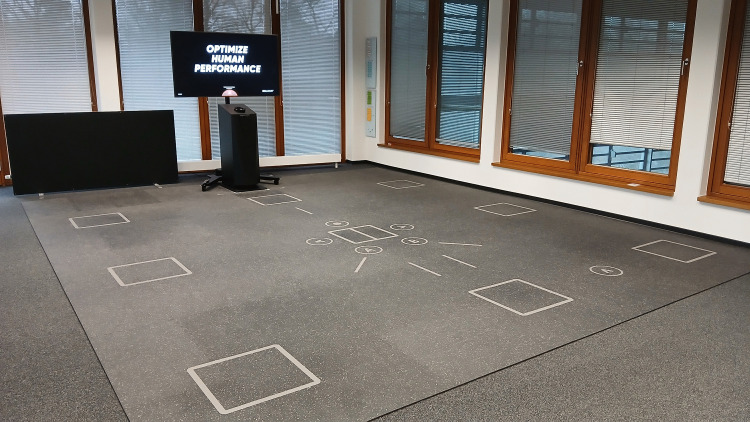
Photograph of the SKILLCOURT setup showing the 65" screen, the central field with five inner target fields (reactive stepping tasks), and eight outer target fields (agility tasks). Published under a Creative Commons Attribution 4.0 International (CC BY 4.0) license with permission from Skillcourt GmbH. Original copyright © 2026 Skillcourt GmbH.

Agility and motor-cognitive tests were assessed using the SKILLCOURT system (SKILLCOURT GmbH, Bergrheinfeld, Germany). Participant position was continuously tracked using LiDAR technology (40 Hz) on a 5 × 5 m rubber mat court with a 65″ screen displaying task-specific cognitive stimuli (Fig 2).

To assess lower-extremity reaction speed, both a simple and a choice reaction task were performed. Participants stood in an active position with slightly flexed knees and feet placed hip-width apart in the centre field of the court. In the *simple reaction test*, participants responded to a series of visual stimuli (an orange rectangle presented at the centre of the screen) by performing rapid reactive sidesteps. The task consisted of 30 consecutive trials, with 15 trials performed using the dominant leg, followed by 15 trials using the non-dominant leg. Response time was defined as the time from stimulus onset to the moment the participant reached the target square with the sidestep, thereby including both reaction and movement execution components. After reaching the target field, participants immediately returned to the center position for the next trial. This procedure was identical to all motor-cognitive stepping tests. Performance was measured as the mean response time (s) across both legs.

Similarly, in the *choice reaction test*, participants responded as quickly as possible to a series of two visual stimuli (an orange or a blue rectangle) presented in random order at the centre of the screen. They stepped to the right inner target field in response to orange rectangles and to the left for blue rectangles. Two blocks of 15 consecutive trials were conducted. For both the simple and choice reaction tests, the interstimulus interval varied randomly between 2 and 5 s. Performance was quantified as mean response time (s) across trials. Each test was performed once.

The assessment of key executive functions (cognitive flexibility, working memory, and interference control [46,47]) consisted of three separate tests, administered in the following order: the task switching task (cognitive flexibility [46]), the 2-back task (working-memory [48]), and a Stroop word-color condition (interference control [47,49]). Tests were performed sequentially in a fixed order, each comprising 30 consecutive stimuli, with a short break of 15–30 sec between tests.

**Fig 3:**
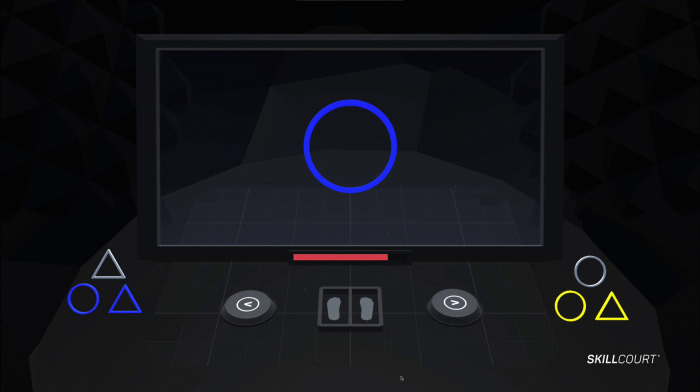
Schematic illustration of the SKILLCOURT task switching test showing the central field with the left and right inner target fields, the screen with stimulus presentation (blue circle), and the legend bar (sidestep to the left target field: blue or white triangle; sidestep to the right target field: yellow or white circle), which was shown throughout the task. Published under a CC BY 4.0 license with permission from Skillcourt GmbH. Original copyright © 2026 Skillcourt GmbH.

In the *task switching test*, participants responded to a series of two types of visual stimuli: shapes (triangle or circle) and colors (yellow or blue). When the shapes were displayed in white, participants reacted based on shape: ‘circle’ required a reactive step to the right and ‘triangle’ a step to the left inner target field. When the shapes were displayed in color, participants reacted based on color only, regardless of shape: ‘yellow’ required a reactive step to the right and ‘blue’ a step to the left (Fig 3). The next stimulus was displayed after participants returned to the centre field. The legend bar was shown throughout the task.

**Fig 4:**
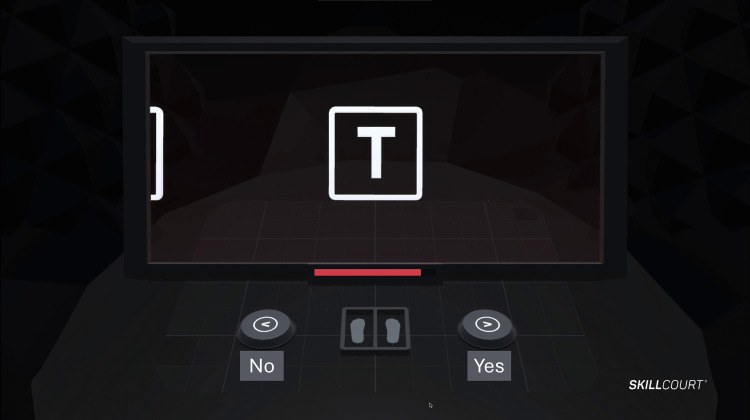
Schematic illustration of the SKILLCOURT 2-back task (B) showing the central field with the left and right inner target fields and the screen with letter stimuli. Participants stepped to the right field if the current stimulus matched the stimulus presented two trials earlier and to the left if it did not. Published under a CC BY 4.0 license with permission from Skillcourt GmbH. Original copyright © 2026 Skillcourt GmbH.

In the *2-back task*, participants viewed a continuous sequence of letters and had to decide whether the current letter matched the one shown two positions earlier (Fig 4). A match required a reactive step to the right and a non-match a step to the left inner target field. The next stimulus was displayed after participants returned to the centre field. If participants made an error, they were instructed to proceed directly to the next stimulus, as the sequence continued without interruption.

**Fig 5:**
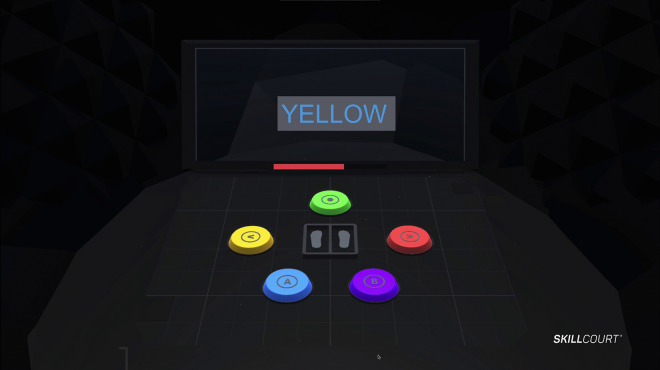
Schematic illustration of the SKILLCOURT Stroop word-color condition showing the central field with the 5 inner target fields and the screen with color-word stimuli. Legend bar displaying the color-directions was shown on the screen throughout the task. Published under a CC BY 4.0 license with permission from Skillcourt GmbH. Original copyright © 2026 Skillcourt GmbH.

In the *Stroop word-color condition*, a series of color words were presented in incongruent font colors. Participants were instructed to respond to the meaning of the word while ignoring the color in which it was displayed. Each word meaning was assigned one target field on the SKILLCOURT (Fig 5). The color-directions were shown throughout the task to reduce task complexity by minimizing memory demands. If participants felt uncomfortable, a member of the test team stood next to them to intervene in case of balance issues. The next stimulus was displayed after participants returned to the centre field.

All three executive function tests were performed once. Test performance was operationalized using the Inverse Efficiency Score (IES) (Eq. 1) following previous studies [35,36].


$$\begin{array}{c} IES=\frac{responsetime(s)}{\left(\text{1}-errorrate\right)} \end{array}$$

Trials from a given test were excluded if error rates exceeded 30% within that test. All remaining data from the respective participant were retained. This procedure was applied to ensure internal validity of the task-specific measures and to reduce the influence of insufficient task performance. Additionally, IES scores were averaged across all three executive function tests to derive a composite executive function score for reliability analysis.

**Fig 6:**
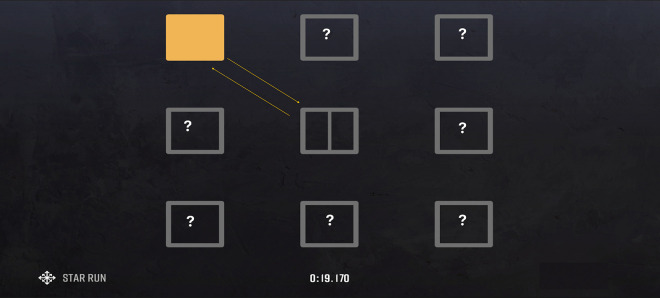
SKILLCOURT Random Star Run agility test with a highlighted outer target field indicating the required movement with returning to the central field for the next stimulus. Published under a CC BY 4.0 license with permission from Skillcourt GmbH. Original copyright © 2026 Skillcourt GmbH.

Agility performance was assessed using the Random Star Run [35,36]. Starting from the centre of the court, participants had to reach one of eight outer target fields, which was visually indicated in yellow on a representation of the court displayed on the screen. After reaching the indicated field, they returned to the centre before the next target was presented. In each trial, participants completed a sequence of eight outer target fields in random order (random permutation without repetition), introducing a decision-making component (Fig 6). Participants were instructed to perform the task as quickly as possible and completed three recorded trials, with a 60-s seated rest between trials. Safety was ensured by having one experimenter closely following the participant to provide support in case of a fall or trip. Performance was measured as total completion time (s), with the best trial (fastest time) used for analysis.

**Fig 7:**
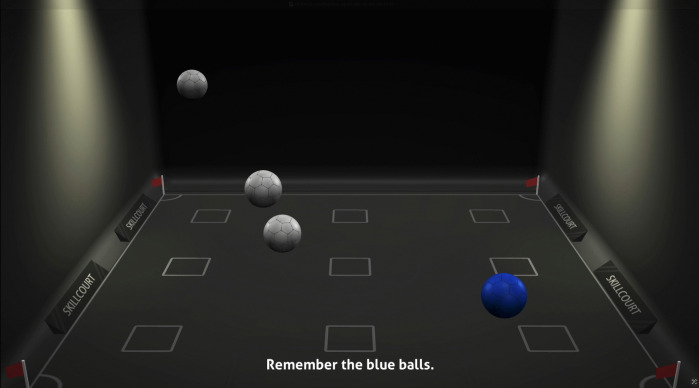
SKILLCOURT dual task agility test (Random Star Run with multiple-object tracking): Court display showing four stationary balls, with one ball highlighted in blue at the start of the task. Published under a CC BY 4.0 license with permission from Skillcourt GmbH. Original copyright © 2026 Skillcourt GmbH.

The dual task test combined reactive agility (Random Star Run) with multiple object tracking. During this test, participants start standing in the centre field while four white balls (foreground) and a representation of the court’s outer target fields (background) were displayed on the screen. At the beginning of each round, one ball was highlighted in blue for 3 seconds (Fig 7).

**Fig 8:**
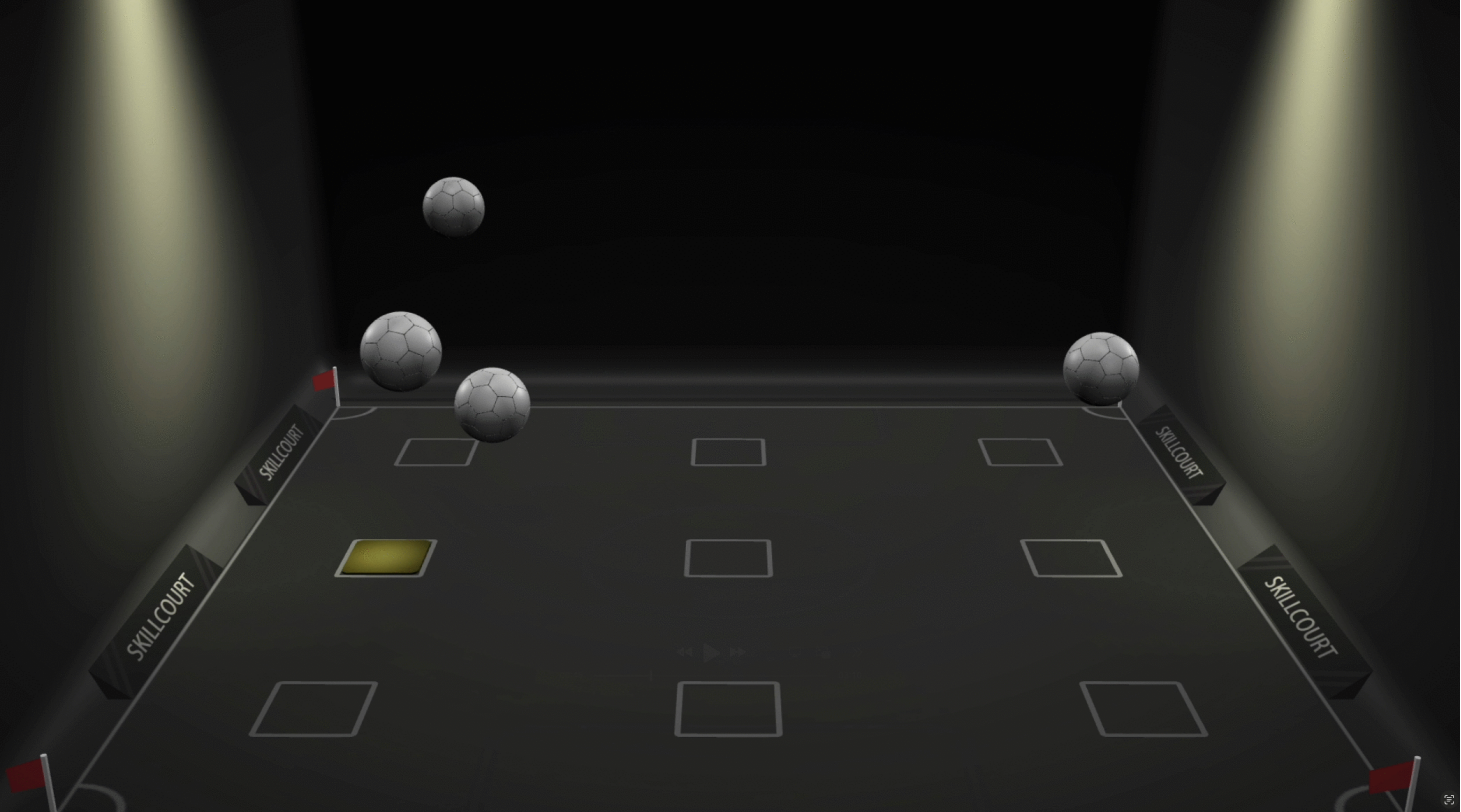
SKILLCOURT dual task agility test (Random Star Run with multiple-object tracking): Moving objects and a highlighted outer target field indicating the required movement direction while participants continue tracking the balls. Published under a CC BY 4.0 license with permission from Skillcourt GmbH. Original copyright © 2026 Skillcourt GmbH.

It then turned white, and balls began moving in a three‐dimensional space. Simultaneously, one of five outer target fields on the court (3 top and 2 middle fields) was highlighted in yellow, indicating that the participant should move to the corresponding field (Fig 8).

**Fig 9:**
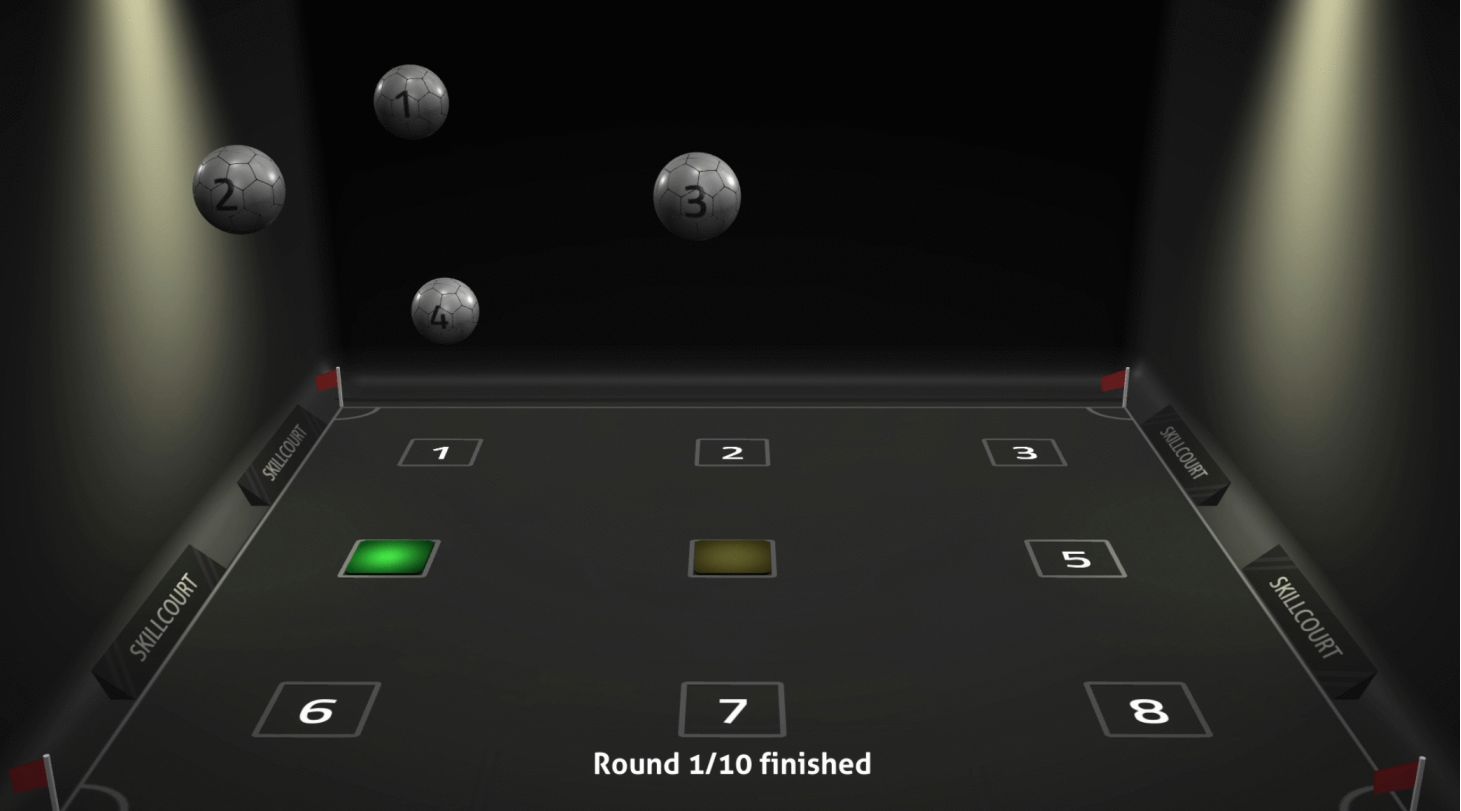
SKILLCOURT dual task agility test (Random Star Run with multiple-object tracking): After 10 s, the four balls stop and are numbered along with the corresponding target fields. Participants are required to step onto the field associated with the ball that was initially highlighted in blue. Published under a CC BY 4.0 license with permission from Skillcourt GmbH. Original copyright © 2026 Skillcourt GmbH.

After reaching the field, the next target field was highlighted in a randomized order. After 10 s, the balls stopped moving, and each ball and field on the court was assigned a number. To select the ball that was initially displayed in blue, participants had to activate the corresponding field (Fig 9).

If the participant correctly identified the target ball, the speed of the moving balls increased for the next trial. The dual task agility test consisted of ten 10‐s trials. Participants were instructed to reach as many fields as possible in each trial (minimum of one) while avoiding losing the target ball. The final score was calculated based on the number of fields reached and ball speed. If the ball was not correctly identified or no field was reached, the score for that trial was set to zero. The final score was obtained by summing the scores across all ten trials.

#### Computer-based cognitive tests.

The computer-based cognitive assessment was conducted using PsychoPy software [50] in a 1:1 setting under standardised, seated conditions (room, lighting, temperature, computer setup, keyboard, and chair). Responses were recorded via keyboard inputs using a German keyboard layout. The cognitive test battery included the same basic reaction time and key executive function (working memory, inhibitory control, and cognitive flexibility [46,47]) tests as the SKILLCOURT tests, performed in the same order. Each task began with a detailed explanation provided by a study team member, followed by up to three practice trials until participants indicated that they had no further questions and felt confident performing the task.

*The simple and choice reaction tests* consisted of 20 trials each, with a randomized interstimulus interval of 2–4 seconds. Participants were instructed to respond as quickly as possible to the appearance of an “X” symbol, either in a single location (simple reaction, index finger) or an orange or blue square on the screen (choice reaction), by pressing one key corresponding to the target (left index finger: “orange”; right index finger: “blue”). In *the 2-back-test,* which reflects mainly working memory capacity [48], participants were shown a continuous sequence of numerical stimuli and were asked to quickly determine whether the current number matched the number presented two trials earlier by pressing key x for “yes” and key y for “no.”

In the *Stroop word–color condition*, which reflects interference control [47,49], color words were displayed sequentially in incongruent font colors. As in the SKILLCOURT test version, participants were instructed to respond to the written word while ignoring the font color. The stimulus-response mapping was kept constant and continuously available to participants, with color-coded response keys “Q” for red, “W” for green, “E” for blue, and “A” for yellow, so that no memorization of key-color associations was required. Both the 2-back and Stroop tests included a total of 64 stimuli with a randomized interstimulus interval.

*The task switching test* on cognitive flexibility [46] required participants to respond to 40 letter-number combination (e.g., A4) by pressing “b” for both consonants and odd numbers or “n” for vowels and even numbers, with the target stimulus switched every second trial [51]. However, this test was too challenging for the present sample, with most participants unable to complete it within an acceptable error rate, and in several cases discontinuing the task. It was therefore removed from the analysis. Consequently, construct validity of the SKILLCOURT task switching test could not be examined.

For all tests, participants were instructed to perform the tests as quickly and as accurately as possible. For the simple and choice reaction tests, the mean reaction time (s) was used as the primary outcome measure. For the executive function tests, only the IES was used. Consistent with the SKILLCOURT tests, trials with error rates above 30% were excluded from the analysis.

#### Motor tests.

Functional mobility was assessed using the *Timed Up and Go (*TUG) test [52], in which participants stood up, walked 3 m, turned, and returned to the chair as quickly and safely as possible. The fastest trial (s) of three was used for analysis. TUG completion time is considered a reliable and valid multicomponent measure, showing moderate to strong correlations with established measures of physical function, including the Berg Balance Scale, gait speed, and activities of daily living [52], as well as fall risk [53].

*Self-selected walking speed* was measured along a 10-m straight walkway using a capacitive pressure platform (60 Hz; Zebris FDM, Zebris Medical GmbH, Isny, Germany) and calculated as the average of 10 consecutive barefoot passes (m/s). The 2-m pressure plate was positioned in the middle of the walkway. Usual walking speed is associated with functional fitness, dynamic balance, and agility [54], with speeds below 1 m/s indicating increased fall risk in older adults [55]. To simulate increased cognitive load during locomotion, both the TUG and the walking speed test were modified by adding a concurrent cognitive subtraction task serving as a distractor, as applied in previous studies [56,57]. Participants received a random number as a start signal for the TUG and at the beginning of each 10-m walking trial. They were instructed to continuously subtract three aloud while walking as quickly and accurately as possible. For both, the fastest of three trials (s) each was used for analysis.

#### Outcome selection for construct validity of the SKILLCOURT.

To examine the convergent construct validity of the SKILLCOURT agility test (Random Star Run), several motor and cognitive measures, including the TUG, self-selected walking speed, PC-based simple and choice reaction time, and the PC-based Stroop word-color condition, were included as predictors (Table 1). These constructs were selected because agility, defined as the ability to perform coordinated, rapid whole-body movements in response to visual stimuli [23], depends on rapid motor execution (including locomotion with changes of direction), perceptual processing speed, response selection, and response inhibition under time-constrained conditions. Accordingly, previous studies have identified change of direction tasks, as well as processing speed and interference control (Stroop word–color condition), as significant predictors of the Random Star Run agility test in athletes [24,25,36].

**Table 1:** Overview of motor and cognitive outcomes correlated with SKILLCOURT tests.

Tests	Outcomes	Tested Ability
SC-Random Star Run (agility)	Time Up and Go (ST)	Functional Mobility
	Walking Speed (ST)	Walking speed
	PC-based simple reaction	Basic processing speed
	PC-based choice reaction	Movement selection
	PC-based Stroop word-color	Interference control
SC-DT agility	Time- Up-and-Go (DT)	Mobility (dual task)
	Walking Speed (DT)	Walking speed (dual task)
	PC-based simple reaction	Basic processing speed
	PC-based choice reaction	Movement selection
	PC-based Stroop word-color	Interference control
	PC-based 2-Back	Working memory)
SC-simple reaction	PC-based simple reaction	Basic processing speed
SC-choice reaction	PC-based choice reaction	Movement selection
SC-2-back	PC-based 2-Back	Working memory
SC-Stroop	PC-based Stroop word-color	Interference control

Abbreviations: SC = SKILLCOURT, ST = single task, DT = dual task, s = seconds, SD = standard deviations

The SKILLCOURT dual task agility task, which combines the Random Star Run with multiple object tracking, involves similar motor demands but likely higher working memory requirements. Therefore, the TUG dual task and dual task walking speed were included as motor predictors, and the PC-based 2-back test was additionally included to account for increased working memory demands (Table 1). Accordingly, previous work has shown that this task is associated with attention, reaction time, and executive functions [24].

The motor-cognitive reactive stepping tasks on the SKILLCOURT were correlated with their computer-based counterparts (Table 1), except for the task switching-test, which was excluded as previously reported due to substantial errors and task discontinuations in the PC-based version. Consequently, construct validity analyses for the SKILLCOURT executive function composite score, which includes all three executive function measures, were not conducted.

### Statistics

#### Intersession reliability.

To analyse potential changes in SKILLCOURT test performances across the three test days, a linear mixed model was applied with test day as a fixed factor and participant ID as a random intercept using Jamovi (Version 2.7.4). Residuals were assessed for normality (Q-Q plots, Shapiro–Wilk test) and homoscedasticity (residuals-versus-predicted plots). Post-hoc comparisons were adjusted for multiple testing using Tukey’s method. Effect sizes were interpreted as small (η² = 0.01), medium (η² = 0.06), and large (η² = 0.14) [58]. As no cognitive exclusion criteria were applied, cognitive function (MoCA) and age were tested as covariates but did not affect the main effects and were therefore not retained. Sensitivity analyses excluding participants with MoCA < 26 (indicative of potential cognitive impairment [39]) yielded comparable results.

To assess relative and absolute reliability, the intraclass correlation coefficient (ICC_3,1_; two-way mixed-effects model, consistency, single measures) and the coefficient of variation (CV, %) were used. ICCs were calculated using a two-way mixed-effects model for single measurements and absolute agreement (Jamovi; Version 2.7.4). Due to missing data particularly at test day 3, ICCs were computed pairwise (days 1–2, 2–3, 1–3). CVs were calculated only for participants with complete data across all test days. ICC scores were classified as follows: poor (< 0.50), moderate (0.50–0.75), good (0.75–0.90), and excellent (≥0.90) reliability [59]. CVs of ≤ 10% were interpreted as excellent, 10–20% as good, 20–30% as acceptable, and > 30% as poor [60]. To evaluate the sensitivity of the SKILLCOURT tests to detect meaningful performance changes (usefulness), the typical error (TE) and smallest worthwhile change (SWC) corresponding to a medium effect size of 0.5 were calculated according to Hopkins et al. 2004 [61]. A test was considered sensitive if the SWC exceeded the TE. TE and SWC were compared for each pairwise combination of test days. CV, TE, and SWC were computed using Microsoft Excel (Office 365).

#### Convergent construct validity.

SKILLCOURT test outcomes (test day 1 only) were correlated with corresponding motor and cognitive tests using bivariate correlation analyses (Pearson or Spearman’s rank correlation). Correlation strength was interpreted as weak (< 0.39), moderate (0.4–0.69), strong (0.7–0.89), and very strong (≥ 0.90) [62].

For each SKILLCOURT test outcome, linear regression analyses were performed using the respective motor and cognitive test performances as potential predictors (Table 1). Enter and stepwise backward models were used to identify the best predictors (Jamovi Version 2.7.4). The alpha level was set at p < 0.05 for all analyses.

Residual normality was assessed using Q–Q plots. Homoscedasticity was evaluated with the Breusch–Pagan test (p > .05) and inspection of residuals versus fitted values. Autocorrelation was examined using the Durbin–Watson test (p > .05), and multicollinearity was assessed using variance inflation factors (all VIFs < 5) [63]. To address violations of homoscedasticity adjusted robust standard errors were used for the SKILLCOURT choice reaction test [64]. Standardised coefficients (β) were interpreted as small (0.1–0.29), medium (0.30–0.49), or large (> 0.50) associations [65]. Analyses were adjusted for age. The potential influence of MoCA performance was tested as a covariate but did not affect the individual final regression models. Similarly, sensitivity analyses excluding participants with lower MoCA scores yielded comparable results. Accordingly, MoCA scores were not retained in the models.

## Results

### Participant characteristics

Of the 80 initially included participants, five were excluded due to severe walking impairments or physical or cognitive limitations. Three additional participants withdrew after the familiarisation session. Thus, data from 72 participants (♀ = 48; age: 73.7 ± 7.6 yrs; range 63–91, body weight: 71.2 ± 14.4 kg, body height: 169 ± 10 cm; body mass index: 24.9 ± 3.8 kg/m^2^; mean ± SD) were included in the final analysis. Participants were predominantly community-dwelling, with 5 living independently in senior residences (Table 2). Mean formal education was 10.7 ± 1.7 years (range: 6–13). High school graduation was reported in 23 participants (32%), while 49 participants (68%) completed secondary or basic secondary school. Twenty-three individuals (32%) reported that they experienced at least one fall in the previous 12 months. One adverse event occurred. One participant experienced an anterior cruciate ligament rupture during the Random Star Run agility test on test day 3. Data from one participant in the SKILLCOURT simple reaction test and from two participants in the choice reaction test were excluded due to implausible outliers.

**Table 2:** Summarises the results of the assessed characteristics.

Characteristic variables	Mean ± SD (min-max)
SF12-Physical (score)	48 ± 8 (28–59)
SF12-Mental (score)	55 ± 6 (35–65)
GDS Depression (score)	1.2 ± 1.4 (0–8)
Moderate physical activity (days/week ≥30 min)	3.1 ± 2.0 (0–7)
FES-I (score)	19.3 ± 3.7 (16–31)
MoCA	27.5 ± 2.5 (21–30)
Hand grip strength (kg)	33.8 ± 9.0 (18–58)
Sit to Stand (number)	15.6 ± 3.6 (9–25)

Abbreviations: GDS = Geriatric Depression 15-Scale, FES-I = Falls Efficacy Scale-International, MoCA = Montreal Cognitive Assessment (MoCA).

### Performance changes across test days for individual SKILLCOURT tests

Except for the SKILLCOURT task switching and choice reaction tests, significant learning effects over time were observed for all tests (Tables 3 and 4). SKILLCOURT Random Star Run, dual task agility, 2-back, Stroop word-color, and the composite executive function test score improved from day 1 to day 2 (p < .05). The simple reaction, Random Star Run, dual task agility, Stroop, and the composite executive function test score improved from day 1 to day 3 (p < .05). No significant differences were observed between test day 2 and 3 for any test (Tables 3 and 4).

**Table 3:** Descriptive data for between-day differences in SKILLCOURT outcomes.

Skillcourt Tests	Outcome	Statistics	Day 1	Day 2	Day 3
Random Star Run	Total time (s)	N	72	70	38
		Mean (95% CI)	33.2 (31.4–34.9)	32.2 (30.4–33.9)	32.9 (30.3–35.4)
Dual task agility	Score (points) Score (points)	N	72	69	38
		Mean (95% CI)	52.2 (43.8–60.6)	64.4 (52.6–76.2)	66.1 (53.5–78.7)
Simple reaction	Reaction time (sec)	N	71	69	39
		Mean (95% CI)	0.71 (0.68–0.73)	0.70 (0.67–0.72)	0.67 (0.65–0.70)
Choice reaction	Response time (s)	N	71	69	39
		Mean (95% CI)	0.87 (0.84–0.90)	0.87 (0.84–0.89)	0.86 (0.82–0.89)
Task switching	Response time – IES (sec)	N	70	64	38
		Mean (95% CI)	1.48 (1.39–1.57)	1.42 (1.33–1.51)	1.49 (1.36–1.62)
2-Back	Response time – IES (s)	N	55	53	30
		Mean (95% CI)	1.98 (1.86–2.10)	1.80 (1.69–1.91)	1.95 (1.76–2.15)
Stroop word-color	Response time – IES (s)	N	70	68	37
		Mean (95% CI)	1.60 (1.54–1.70)	1.55 (1.45–1.65)	1.50 (1.38–1.62)
Composite executive function score	Response time – IES (s)	N	53	46	29
		Mean (95% CI)	1.65 (1.56–1.73)	1.50 (1.41–1.59)	1.57 (1.46–1.68)

Abbreviations: sec = seconds, IES = Inverse Efficiency Score, CI = confidence intervals

**Table 4:** Linear mixed-effects model results for between-day differences in SKILLCOURT outcomes.

Skillcourt Tests	Main fixed effects		Post hoc comparisons
Random Star Run	F_107_ = 5.26, p = .007, η² = 0.09^1^		M1 vs. M2: t_106_ = 3.03, p = .009
Dual task agility	F_109_ = 5.63, p = .005, η² = 0.09^2^		M1 vs. M2: t_107_ = −3.06, p = .008 M1 vs. M3: t_111_ = −2.47, p = .039
Simple reaction	F_107_ = 4.49, p = .013, η² = 0.08^3^		M1 vs. M3: t_108_ = 2.89, p = .013
Choice reaction	F_108_ = 0.34, p = .715, η² = 0.01		
Task switching	F_100_ = 0.66, p = .518, η² = 0.01		
2-Back	F_76_ = 3.90, p = .025, η² = 0.09^4^		M1 vs. M2: t_73_ = 2.79, p = .018
Stroop word-color	F_103_ = 3.56, p = .032, η² = 0.07^5^		M1 vs. M3: t_104_ = 2.40, p = .047
Composite executive function score	F_65_ = 7.84, p < .001, η² = 0.19^6^		M1 vs. M2: t62 = 3.58, p = .002 M1 vs. M3: t67 = 2.86, p = .016

### Intersession Reliability and Usefulness of individual SKILLCOURT tests

Moderate to good relative reliability across all three test days was observed for the SKILLCOURT dual task agility, simple reaction, and Stroop word-color, as well as the composite executive function test score (ICC: 0.70 to 0.89), with the Random Star Run showing excellent reliability (ICC: 0.93–0.94; (Table 5).

**Table 5:** Intraclass correlation coefficients (ICC) and usefulness for pairwise comparisons between test days for all SKILLCOURT outcomes.

Skillcourt Tests	Outcome	Days (N)	ICC (95% CI)	TE	SWC (0.5)
Random Star Run	Total time (s)	1–2 (70)	0.93 (0.89–0.95)	2.0	3.8
		2–3 (38)	0.93 (0.88–0.97)	2.1	3.8
		1–3 (38)	0.94 (0.88–0.97)	2.0	3.8
Dual task agility	Score (points)	1–2 (69)	0.70 (0.56–0.80)	24.0	18.2
		2–3 (38)	0.75 (0.56–0.86)	21.9	25.0
		1–3 (38)	0.76 (0.59–0.87)	19.3	18.2
Simple reaction	Response time (s)	1–2 (68)	0.80 (0.69–0.87)	44.4	53.2
		2–3 (37)	0.78 (0.61–0.88)	44.6	45.6
		1–3 (38)	0.71 (0.50–0.84)	53.4	53.2
Choice reaction	Response time (s)	1–2 (68)	0.79 (0.67–0.86)	49.7	56.6
		2–3 (37)	0.49 (0.20–0.70)	70.1	51.7
		1–3 (38)	0.66 (0.44–0.81)	63.2	56.6
Task switching	Response time – IES (s)	1–2 (62)	0.65 (0.58–0.78)	221.1	195.4
		2–3 (33)	0.83 (0.68–0.91)	173.9	183.0
		1–3 (36)	0.64 (0.40–0.80)	253.1	195.4
2-Back	Response time – IES (s)	1–2 (45)	0.74 (0.57–0.85)	201.1	221.8
		2–3 (22)	0.76 (0.51–0.89)	200.7	196.0
		1–3 (23)	0.64 (0.32–0.83)	291.8	221.8
Stroop word-color	Response time – IES (s)	1–2 (66)	0.89 (0.83–0.93)	138.0	204.8
		2–3 (35)	0.83 (0.69–0.91)	165.3	210.1
		1–3 (36)	0.82 (0.67–0.90)	156.3	204.8
Composite executive function score	Response time – IES (s)	1–2 (43)	0.85 (0.75–0.92)	108.7	161.3
		2–3 (19)	0.87 (0.69–0.95)	91.6	150.1
		1–3 (20)	0.82 (0.60–0.92)	115.2	161.3

Abbreviations: IES = Inverse efficiency score, N = number of participants, ICC = Intraclass correlation coefficient, TE = Typical error, SWC = smallest worthwhile change, CI = confidence interval.

The SKILLCOURT choice reaction test showed good reliability between test days 1 and 2 (ICC = 0.79), with lower but still moderate reliability across longer intervals (ICC = 0.49–0.66). The task switching test showed good reliability between test days 2 and 3 (ICC: 0.83) with lower but still moderate reliability between test days 1 and 2 (ICC: 0.65) and test days 1 and 3 (ICC: 0.64). The 2-back demonstrated good reliability between all test days (ICC 0.74–0.76), except between test days 1 and 3, which yielded moderate reliability (ICC: 0.64; Table 5).

Sufficient sensitivity for performance changes can be assumed for the SKILLCOURT Random Star Run, simple reaction, Stroop word-color, and the composite executive function test score, with the SWC exceeding the TE for all test day comparisons. For the choice reaction and 2-back tests, this applied only to the comparison between test days 1 and 2, and for the task switching and dual task agility only to the comparison between test days 2 and 3 (Table 5)

Excellent absolute reliability was observed for the Random Star Run, simple and choice reaction, Stroop word-color, and the composite executive function test score (CV: ~ 5–7%), higher but still acceptable CVs for the task switching and 2-back tests (CV: ~ 12%), and poor reliability for the dual task agility test (CV: ~ 31%; Table 6).

**Table 6:** Coefficient of variation (CV) across the three test days for participants with complete data (mean CV calculated across the three measurement time points per participant) for all SKILLCOURT outcomes.

Skillcourt Tests	Outcome	N	CV ± SD (%)
Random Star Run	Total time (s)	38	4.6 ± 2.8
Dual task agility	Point Score	38	31.4 ± 19.8
Simple reaction	Reaction time (s)	36	6.0 ± 4.1
Choice reaction	Reaction time (s)	37	6.1 ± 3.7
Task switching	IES (s)	32	11.7 ± 7.1
2-Back	IES (s)	20	12.0 ± 6.6
Stroop word-color	IES (s)	34	7.2 ± 3.4
Composite executive function score	IES (s)	18	7.0 ± 3.5

Abbreviations: IES = Inverse efficiency score, N = number of participants, CV = coefficient of variation, SD = standard deviation

### Convergent Construct Validity of individual SKILLCOURT tests

Table 7 presents the descriptive statistics of the SKILLCOURT tests and their corresponding motor and cognitive outcome measures.

**Table 7:** Descriptive statistics of the SKILLCOURT, motor, and cognitive tests.

Measure	Unit	Mean ± SD (min–max)
SKILLCOURT measures		
SC – Random Star Run	s	33.2 ± 7.6 (21–53)
SC – Dual task agility	points	52.2 ± 36.3 (5–184)
SC – Simple reaction	s	0.71 ± 0.11 (0.51–0.96)
SC – Choice reaction	s	0.87 ± 0.11 (0.65–1.25)
SC – 2-back (IES)	s	1.98 ± 0.44 (1.15–2.71)
SC – Stroop (IES)	s	1.60 ± 1.46 (1.11–3.12)
Motor measures		
Timed Up and Go (ST)	s	6.4 ± 1.4 (3.6–10.5)
Timed Up and Go (DT)	s	7.7 ± 2.6 (3.7–18.1)
Walking speed (ST)	m/s	1.3 ± 0.2 (0.9–1.6)
Walking speed (DT)	m/s	1.1 ± 0.2 (0.53–1.56)
Cognitive measures (PC-based)		
Simple reaction	s	0.34 ± 0.06 (0.23–0.61)
Choice reaction	s	0.61 ± 0.10 (0.43–0.86)
Stroop word–color (IES)	s	1.04 ± 0.28 (0.71–2.22)
2-back (IES)	s	1.28 ± 0.24 (0.87–1.97)

Abbreviations: SC = SKILLCOURT, IES = Inverse efficiency score, ST = single task, DT = dual task.

**Fig 10:**
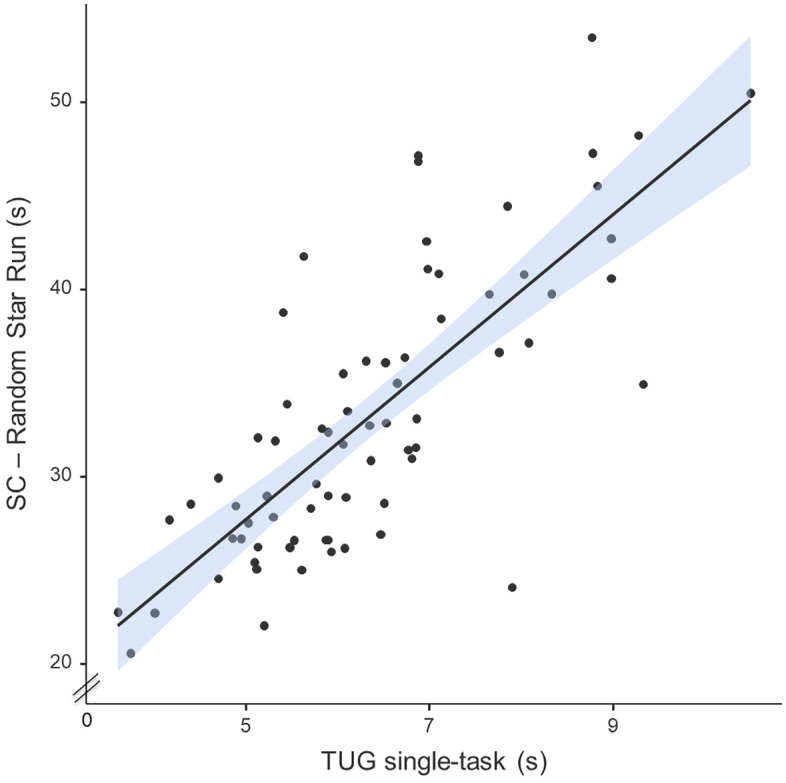
Correlations between SKILLCOURT Random Star Run agility test and the TUG single task.

**Fig 11:**
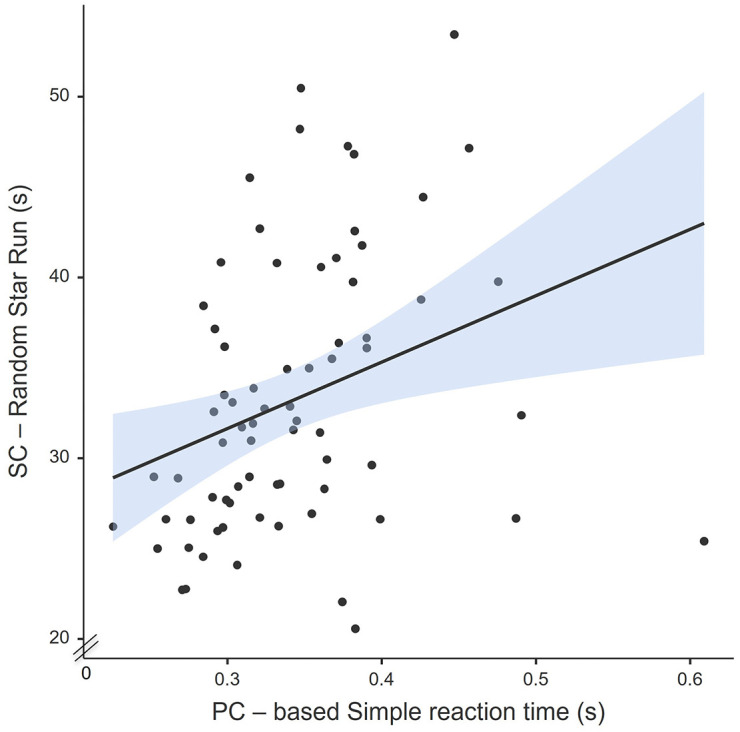
Correlations between SKILLCOURT Random Star Run agility test and the PC-based simple reaction test., and Stroop word-color test.

**Fig 12:**
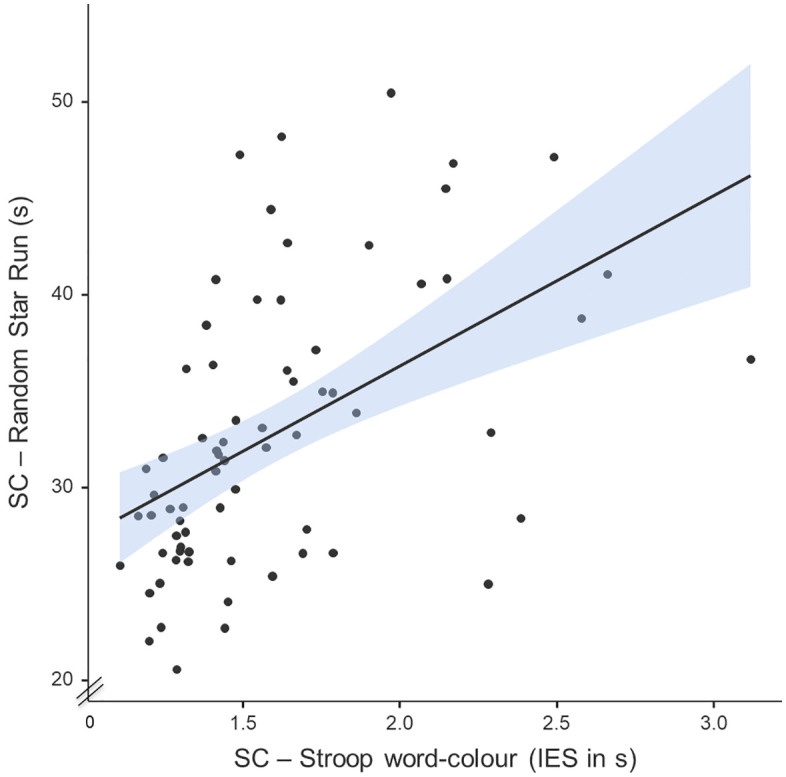
Correlations between SKILLCOURT Random Star Run agility test and the PC-based Stroop word-color test.

Spearman correlations indicated significant relationships between the SKILLCOURT Random Star Run agility and the TUG (r_70_ = 0.75, p < .001), walking speed (r_65_ = −0.53, p < .001), PC-based simple (r_70_ = 0.41, p < .001), PC-based choice reaction (r_68_ = 0.28, p = .018), and the PC-based Stroop word-color test (r_66_ = 0.61, p < .001), as well as age (r_70_ = 0.83, p < .001). A multiple linear regression including all variables yielded a model with good predictive power for the Random Star Run (R^2^ = 0.77; Table 8). Age-adjusted stepwise backward regression identified the TUG single task, PC-based simple reaction test, and PC-based Stroop word-color as the only significant predictors (Table 8). The corresponding associations are illustrated in scatterplots (Figs 10, 11 and 12).

**Table 8:** Results of the linear regression analyses examining concurrent and construct validity of the SKILLCOURT agility tests (adjusted for age).

SKILLCOURT Agility Tests	Enter model
Random Star Run	Main model: R^2^_6, 55_ = 0.77, Adjusted R^2^ = 0.74, AIC = 350, BIC = 367, F = 30.6, p = < .001 Predictors: -TUG-ST: B = 1.89, β = 0.35, SE = 0.49, t = 3.85, p < .001 -SRT-PC: B = 10.33, β = 0.09, SE = 8.23, t = 1.26, p = .215 -Stroop word-color-PC: B = 4.91, β = 0.18, SE = 2.01, t = 2.44, p = .018 -CRT-PC: B = 6.02, β = 0.08, SE = 6.06, t = 0.99, p = .325 -Walking speed-ST: B = −1.10, β = −0.02, SE = 3.79, t = −0.29, p = .774
	**Stepwise backward model**
	Main model: R^2^_4, 63_ = 0.77, adjusted R^2^ = 0.75, AIC = 377, BIC = 390, F = 51.7, p = < .001 Predictors: -TUG (ST): B = 1.91, β = 0.37, SE = 0.44, t = 4.37, p < .001 -Stroop word-color-PC: B = 5.20, β = 0.20, SE = 1.84, t = 2.82, p = .006 -SRT-PC: B = 15.49, β = 0.14, SE = 7.07, t = 2.19, p = .032
Dual task agility (Point score)	**Enter model**
	Main model: R^2^_7, 39_ = 0.46, Adjusted R^2^ = 0.38, AIC = 482, F = 5.90, p = < .001 Predictors: -TUG-DT: B = −9.45, β = −0.42, SE = 3.45, t = −2.74, p = .009 −2-Back-PC: B = −38.72, β = −0.25, SE = 19.75, t = −1.96, p = .057 -Stroop word-color-PC: B = −36.59, β = −0.20, SE = 25.59, t = −1.43, p = .161 -Walking speed-DT: B = −35.52, β = −0.20, SE = 27.14, t = −1.31, p = .198 -CRT-PC: B = −53.98, β = −0.13, SE = 55.17, t = −0.98, p = .334 -SRT-PC: B = 9.64, β = 0.02, SE = 73.78, t = 0.13, p = .897
	**Regression (stepwise backward model)**
	Main model: R^2^_3, 51_ = 0.37, adjusted R^2^ = 0.34, AIC = 537, BIC = 547, F = 10.2, p = < .001 Predictors: -TUG-DT: B = −6.41, β = −0.31, SE = 2.78, t = −2.33, p = .024 −2-Back-PC: B = −44.40, β = −0.29, SE = 17.28, t = −2.57, p = .013

Abbreviations: TUG = Timed Up and Go; ST = single task; DT = dual task; SRT = simple reaction time; CRT = choice reaction time; PC = computer.

**Fig 13:**
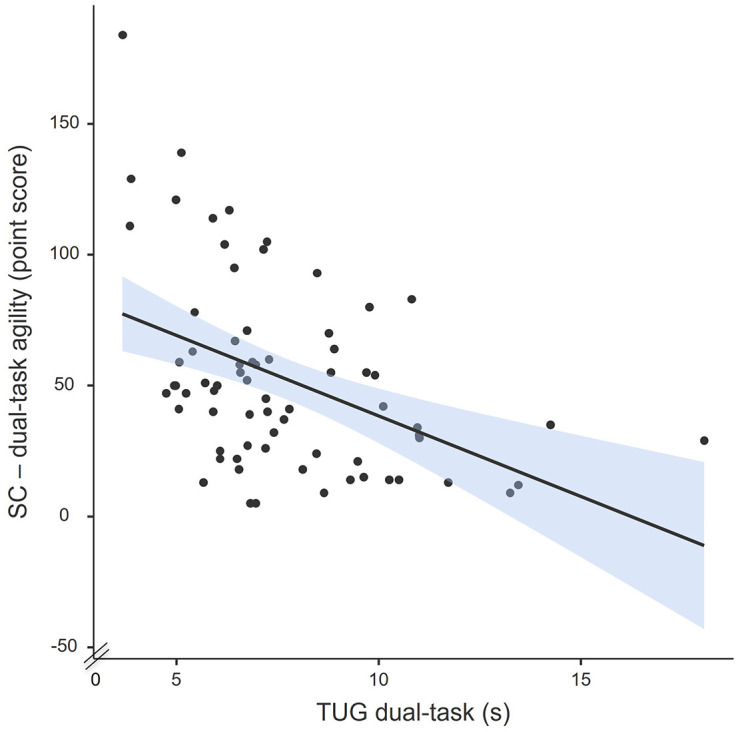
Correlations between the dual task agility test and the TUG dual task.

**Fig 14:**
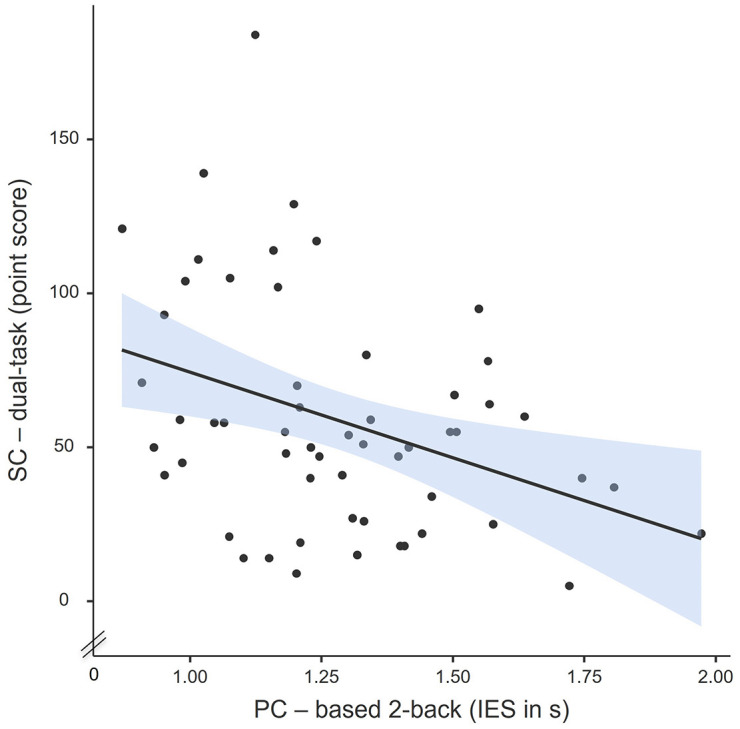
Correlations between the dual task agility test and the PC-based 2-back test.

The SKILLCOURT dual task agility showed significant associations with the dual task TUG test (r_69_ = −0.44, p = < .001), dual task walking speed (r_62_ = 0.34, p = < .007), PC-based simple reaction (r_70_ = −0.33, p = .004), the PC-based executive function tests (2-back: r_54_ = −0.33, p = .013; Stroop word-color: r_66_ = −0.54, p = < .001), and age (r_70_ = −0.56, p < .001) but not with the PC-based choice reaction test (r_68_ = −0.19, p = .109). The enter regression model yielded moderate predictive power (R^2^ = 0.46, Table 8). Stepwise backward regression identified the TUG dual task and the PC-based 2-back test as the only significant predictors (Table 8). The corresponding associations are illustrated in scatterplots (Figs 13 and 14).

**Fig 15:**
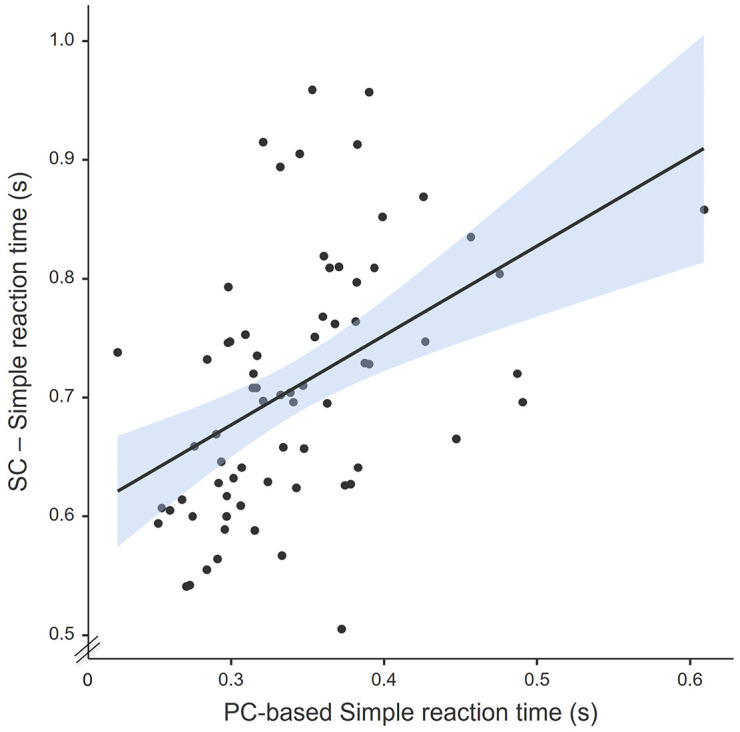
Correlations between the SKILLCOURT and PC-based simple reaction test. The solid black line represents the regression line, and the blue lines indicate the 95% confidence intervals.

**Fig 16:**
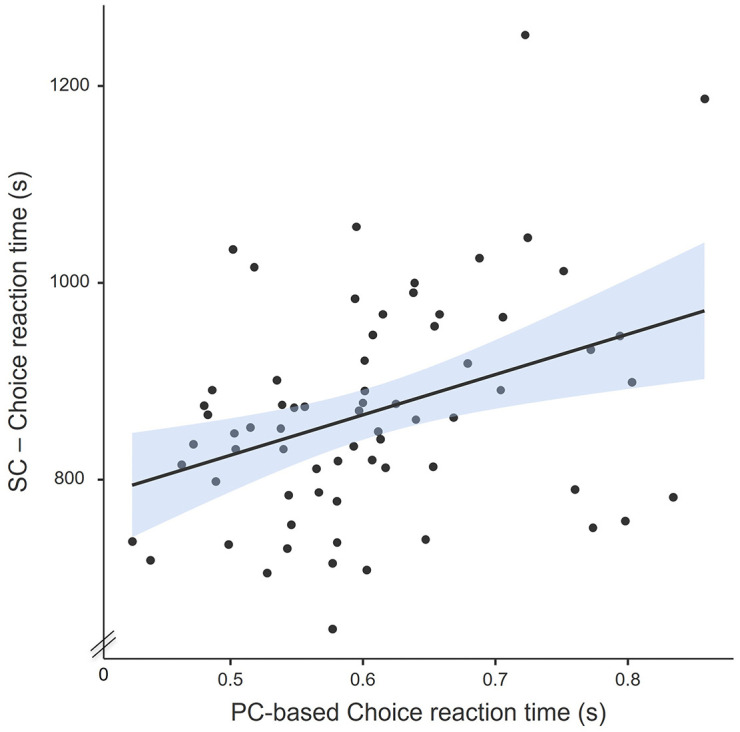
Correlations between the SKILLCOURT and PC-based choice reaction test. The solid black line represents the regression line, and the blue lines indicate the 95% confidence intervals.

**Fig 17:**
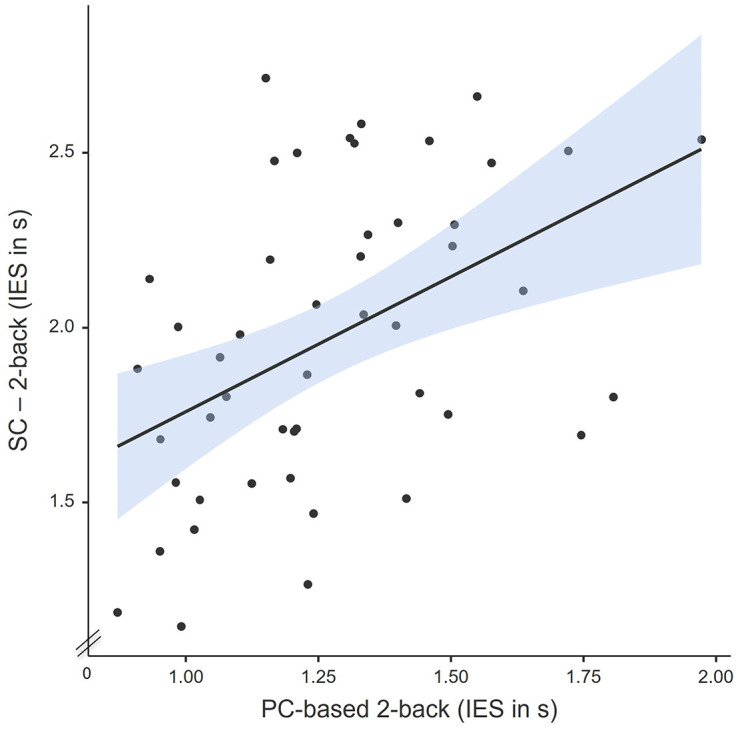
Correlations between the SKILLCOURT and PC-based 2-back test. The solid black line represents the regression line, and the blue lines indicate the 95% confidence intervals.

**Fig 18:**
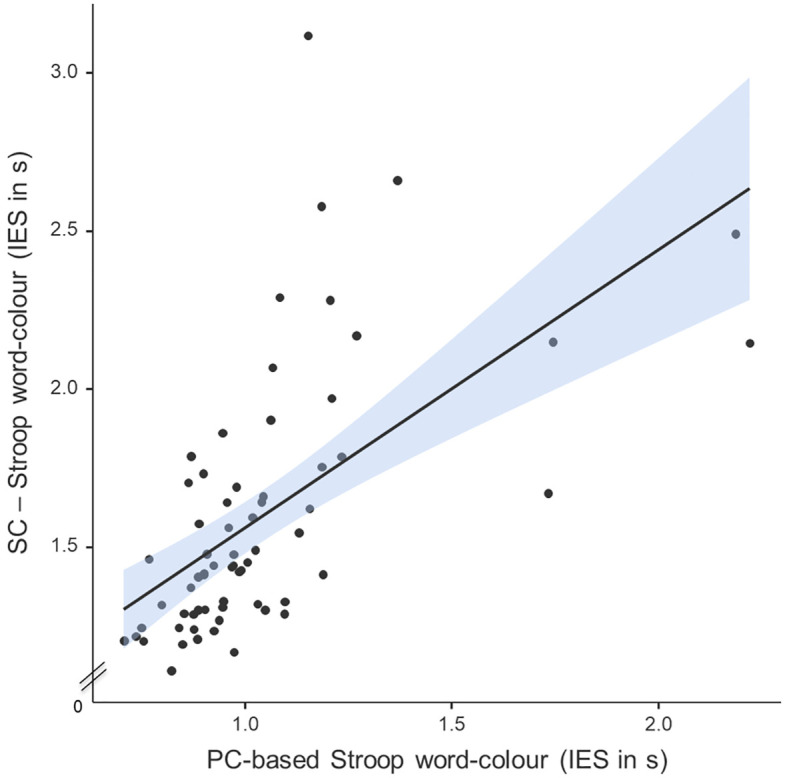
Correlations between the SKILLCOURT and PC-based Stroop word color test. The solid black line represents the regression line, and the blue lines indicate the 95% confidence intervals.

The SKILLCOURT reactive stepping tests in response to simple reaction, choice reaction, and 2-back stimuli showed weak to moderate correlations with their corresponding PC-based cognitive tests (r = 0.34–0.46; Figs 15, 16 and 17). The SKILLCOURT Stroop word-color test demonstrated a moderate to strong correlation with the PC-based version (r = 0.68; Fig 18). All tests showed weak to moderate associations with age (r = 0.22–0.47).

Age-adjusted multiple regression analyses showed that the PC-based tests were significant predictors for all corresponding SKILLCOURT tests (R^2^ = 0.23 to 0.43; Table 9).

**Table 9:** Results of the linear regression analyses examining concurrent validity of the SKILLCOURT basic reaction time and executive function tests (age-adjusted).

SKILLCOURT Motor-cognitive stepping tests	Enter model
Simple reaction	Main model: R^2^_2 68_ = 0.23, adjusted R^2^ = 0.20, AIC = 128, BIC = 119, F = 9.9, p = < .001 Predictors: SRT-PC: B = 0.71, β = 0.43, SE = 0.18, t = 3.92, p < .001
Choice reaction	Main model: R^2^_2,66_ = 0.25, adjusted R^2^ = 0.23, AIC = 119, BIC = 110, F = 11.0, p = < .001 Predictors: CRT-PC: B = 0.35, β = 0.31, SE = 166.3, t = 2.13, p = .037
Stroop word-color	Main model: R^2^_2,63_ = 0.43, adjusted R^2^ = 0.41, AIC = 39.7, BIC = 48.5, F = 23.5, p = < .001 Predictors: Stroop word-color-PC: B = 0.69, β = 0.48, SE = 0.16, t = 4.42, p < .001
2-Back	Main model: R^2^_2,45_ = 0.32, adjusted R^2^ = 0.29, AIC = 42.9, BIC = 50.4, F = 10.4, p = < .001 Predictors: 2-Back-PC: B = 0.65, β = 0.39, SE = 0.21, t = 3.06, p = .004

Abbreviations: sec = seconds; IES = Inverse efficiency score, TUG = Timed Up and Go; SRT = simple reaction time; CRT = choice reaction time; PC = computer, SC = SKILLCOURT.

## Discussion

This study examined the intersession reliability, convergent construct validity, and usefulness of an agility and motor–cognitive stepping test battery on the SKILLCOURT device in functionally independent older adults. Overall, the findings demonstrated good to excellent absolute reliability and usefulness for most tests and day-to-day comparisons, while relative reliability was more heterogenous. Learning effects were observed for most assessments highlighting the importance of sufficient familiarization. The Random Star Run agility test showed good construct validity, while motor-cognitive stepping assessments appear to capture performance aspects less represented in established seated cognitive-only tests, potentially highlighting the contribution of motor–cognitive interactions.

### Performance changes across test days for individual SKILLCOURT tests

Except for the choice reaction and task switching tests, significant improvements in performance were observed for all SKLLCOURT tests relative to test day 1. These improvements likely reflect learning due to familiarisation with the test procedures, improved task understanding and strategy development [66]. The results are consistent with a previous study in recreational athletes [35] for comparable agility and motor-cognitive stepping tests with varying cognitive demands.

Like the previous study [35], no significant learning effects were observed from test day 2 onwards, suggesting that participants had reached a more stable level of performance after the initial exposure. Notably, in contrast to the present study, that study did not include a separate familiarisation session, as testing commenced immediately at the first appointment. While a single exposure may be sufficient for younger or athletic populations, the present findings, showing learning effects after test day 1, suggest that older adults may require more extensive familiarisation and practice trials prior to formal testing [67].

### Intersession reliability and usefulness of individual SKILLCOURT tests

The SKILLCOURT Random Star Run, simple reaction, Stroop word-color, and the composite executive function score, calculated as the average of all three subtests (Stroop word-color, task switching, and 2-back) demonstrated good to excellent absolute (CV: 4.6–7.2%) and relative intersession reliability (ICC: 0.70–0.94). Sensitivity to performance changes was confirmed across all test-day comparisons (SWC > TE), despite group-level learning effects in some comparisons, suggesting parallel absolute performance gains without substantial changes in rank order (ICC).

The observed ICC values are consistent with those reported for comparable agility tests in older adults, such as the functional reactive agility test (ICC = 0.88–0.94) [29]. In this test, participants performed rapid lateral movements to the right or left in response to a randomly delayed visual stimulus indicating movement direction, like the Random Star Run, which involved multidirectional movements. Similarly, the Agility Challenge for the Elderly (ACE) test involves stop-and-go movements, directional changes, and turning during walking has shown comparable ICC values (0.84–0.94) [22]. The Trail-Walking-Test has demonstrated ICC values of 0.83–0.97 in older adults [20]. This test mimics the design of the paper-based Trail-Making-Test (TMT) Part A, requiring participants to move sequentially from flag number 1 to flag number 15, which are placed in random order within a 5 x 5-meter area as quickly and accurately as possible. Although the latter two tests share similarities with the Random Star Run, they lack a reactive decision-making component.

Similar findings were observed for the motor–cognitive stepping tests. For example, the Stepping TMT is conducted on a 1 x 1 m rubber mat with 16 numbered squares arranged in a randomized pattern, with odd numbers on the left side and even numbers on the right. Participants are required to step on the corresponding numbered squares in ascending order as quickly and accurately as possible, resulting in ICC values of 0.81–0.93 [20,68]. Overall, this test is conceptually comparable to the SKILLCOURT stepping tests, as it combines multidirectional stepping with cognitive processing. In contrast to the Stepping-TMT, which follows a predefined stepping sequence with visuospatial demands, the motor–cognitive SKILLCOURT tests also require reactive stepping in response to more complex executive function stimuli, in line with previous research [14,32]. Overall, this suggest that the above-mentioned SKILLCOURT tests provide a level of reliability comparable to similar motor-cognitive assessments in older adults.

For individual assessments, reliability varied depending on the comparison between test days. Only moderate relative reliability was observed for the choice reaction task when comparing test days 2–3 and 1–3 (ICC: 0.49–0.66), for the task switching task when comparing test days 1–2 and 1–3 (ICC: 0.64–0.65), and for the 2-back task for the longest interval (test days 1–3; ICC: 0.64). For these comparisons, sensitivity to detect performance changes could not be confirmed (SWC < TE). Nevertheless, all remaining inter-day comparisons demonstrated good relative reliability for these specific tasks, with higher absolute intra-individual variability across test days only observed for task switching and 2-back (~12%).

Notably, the composite executive function score (average of all three subtests) appeared to reduce single task-specific variability and demonstrated high ICCs, low CVs, and consistently high sensitivity to detect performance changes across test days (SWC > TE). This suggests that, despite moderate variability in individual executive function measures (task switching and 2-back), the composite score seem less affected by task-specific fluctuations and provides a more robust and reliable estimate than single-task outcomes, particularly task-switching and 2-back measures.

Nevertheless, the slightly lower ICC values for these two tasks may reflect individual differences in familiarization and understanding of these relatively complex tasks on test day 1. This aligns with a previous reliability study in recreational athletes [35], which suggested that cognitively more demanding motor–cognitive tests may exhibit greater variability in relative rank order, particularly while task execution strategies and task understanding are still stabilizing. The longer three-month test interval may also be influenced by different consolidation processes, as indicated by the lower ICC values for the 2-back. Such individual trajectories, where some participants improve due to refined strategies, while others show temporary declines due to incomplete consolidation or day-to-day fluctuations, may alter rank order without necessarily affecting group mean performance.

The lower ICC values for the choice reaction test may reflect its relatively higher motor demands, including reactive stepping and dynamic balance, compared to the simple reaction test performed from a fixed stance and the executive function tests, which are primarily cognitive [36]. Due to longer processing times, the latter allow more time for movement responses, potentially making relative rank order less susceptible to minor motor–cognitive fluctuations than in the choice reaction test.

### Convergent construct validity of individual SKILLCOURT tests

Age-adjusted stepwise backward regression identified the TUG, PC-based simple reaction time, and Stroop word-color performance as significant predictors of Random Star Run performance. This is plausible, as the TUG is an established multicomponent mobility test in older adults, which also includes elements of change of direction (primarily turning) [52], although to a lesser extent. Simple reaction time is required for rapid responses to the yellow target field stimuli (basic information processing), while Stroop performance reflects interference control necessary to inhibit inappropriate responses (inhibitory control) and adjust ongoing movements under time-constrained demands. This model demonstrated high predictive power (R^2^ = 0.77), supporting the construct validity of the Random Star Run as an integrated mobility-agility assessment with reactive and executive function components. This is in line with previous studies identifying change of direction performance, processing speed, and interference control as significant determinants of the Random Star Run agility performance in athletes [24,25,36]. However, it cannot be completely ruled out that single outlying data points in the PC-based Stroop word-color task in our dataset influenced the strength of the observed associations.

Regarding the motor-cognitive stepping tests, the SKILLCOURT Stroop word-color task showed moderate to strong correlations with the corresponding PC-based test, supporting its construct validity. Correlations were only moderate for the SKILLCOURT simple reaction and 2-back tests, and weak for the choice reaction test. However, age-controlled linear regression analyses showed that each PC-based cognitive test significantly predicted performance on the corresponding SKILLCOURT test. Predictive power was low to moderate (R^2^ = 0.20–0.41), indicating that performance reflects combined cognitive and motor contributions, with motor-cognitive interactions likely attenuating correlation strength [36]. Balance control and locomotion require both motor and cognitive components [69], whereas PC-based tests performed in a seated position rely primarily on cognitive processes. This aligns with the proposed framework of structural (generic) interference, in which concurrent motor demands and higher-order cognitive control processes share overlapping neural resources [19], thereby reducing correspondence with isolated cognitive assessments. The cognitive resources required to control posture and balance on the SKILLCOURT, especially when alternating motor responses between the left and right leg, may therefore interfere with task-specific cognitive processing and reduce correlations with computer-based assessments [36]. This may explain why previous studies also reported only moderate correlations between motor-cognitive stepping tests and cognitive-only tests (r = 0.31–0.68) even in younger recreational athletes [36,70] but also in older adults [68].

This pattern, as proposed by Hülsdünker et al. [36], can largely be explained by the relative contributions of motor and cognitive components to each SKILCCOURT test. The choice reaction tests involve a substantial motor component and relatively low cognitive load, potentially leading to greater motor–cognitive interference and weaker correlations with the PC version. In contrast, the simple reaction test has lower motor demands (as one foot remains on the ground for all responses), resulting in potentially less interference and slightly higher correlations. SKILLCOURT Stroop word-color condition demonstrated the best model fit among the motor-cognitive measures, showing the strongest correlations with the corresponding PC-based version. In contrast to the reaction time tests, the Stroop test involves higher cognitive demands due to complex stimulus-response mapping and inhibitory control requirements. Although the test also requires coordinated stepping responses across multiple response locations (5 target fields), its primary emphasis is on executive processing rather than motor execution, potentially resulting in a higher cognitive-to-motor demand ratio compared with the choice and simple reaction tests. Nevertheless, the R^2^ value of 0.43 still indicates greater motor contributions and task interference compared with PC-based assessments.

The 2-back test, however, does not fully follow this pattern. Although it is predominantly cognitive, correlations were only moderate. This may reflect differences in task demands, as approximately one fourth of participants test values were excluded from the SKILLCOURT version due to error rates above 30%, whereas only a few exceeded this threshold in the PC-based test, indicating unequal levels of difficulty between the two versions. In addition, while the other SKILLCOURT and corresponding PC-based tests were comparable in stimulus presentation, the 2-back task differed in stimulus format (letters vs. numbers), which may have further limited direct comparability. These findings suggest that both motor demands and task equivalence may critically influence the strength of correlations, with higher cognitive load and greater task similarity promoting stronger relationships.

### Generalizability and sample characteristics

The present findings apply to functionally independent, community-dwelling older adults aged 63–91 years (mean age ~ 74 years). Participants were relatively well educated (approximately 11 years of formal education, with about one third having completed high school). Group means indicate that participants were generally physically healthy and largely free from clinically relevant depressive symptoms. This is supported by SF-12 scores slightly above 50, corresponding to the normative 50th percentile [37], and Geriatric Depression Scale (GDS-15) scores below 6, indicating no depression [38]. Some participants had MoCa scores below 26, suggesting mild cognitive impairment [39]. However, as no cognitive exclusion criteria were applied, sensitivity analyses excluding these individuals, as well as models including the MoCa score as a covariate, did not meaningfully alter the results, indicating robustness with respect to potential cognitive deficits. This may be attributable to the application of performance-based exclusion criteria, where test values with error rates exceeding 30% were excluded to minimize the influence of poor cognitive or motor performance and to preserve internal validity. Nevertheless, the sample cannot be considered entirely cognitively healthy, but rather functionally independent. Average physical activity levels were moderate but slightly below international recommendations, which suggest at least 30 minutes of moderate physical activity on most days of the week [71]. Fear of falling was low to moderate, with only scores above 23 indicating high concern [72]. Approximately 30% of participants reported at least one fall in the previous year, which is consistent with previous reports of about 29% in community-dwelling adults aged 65 years and older [73]. Functional strength measures were slightly above average. Normative values for this age group are approximately 34 kg for men and 23 kg for women for handgrip strength [74], and about 12–14 repetitions in the 30-s sit-to-stand [75], indicating a generally good level of functional health. However, relatively wide ranges and confidence intervals across most measures, including the motor-cognitive SKILLCOURT outcomes, point to a heterogeneous sample with substantial variability.

### Limitations

Despite relatively unremarkable group means, the wide range observed across most participant characteristics indicates substantial interindividual heterogeneity. Due to the limited sample size, these variables could not be considered as additional covariates in the regression models nor examined in moderator or subgroup analyses. Age was considered the most salient and theoretically relevant confounder and was therefore controlled in all validity analyses. Although a significant influence of MoCA was not confirmed, variability in cognitive functioning may still have contributed to interindividual differences in performance on the SKILLCOURT and cannot be fully excluded. Furthermore, intrasession reliability was not assessed, as for most SKILLCOURT tests only a single actual test trial was performed to avoid physical and mental fatigue and to limit the duration of the test days, given that the overall test battery was already extensive. Only around half of participants attended test day 3. Reliability estimates including this day were calculated only for participants with data at both relevant time points, which may have reduced estimate precision.

Several test-specific limitations should also be acknowledged. The dual task agility test showed good relative reliability across test days (ICC: 0.70–0.76) and moderate construct validity, with the TUG dual task and PC-based 2-back performance emerging as the most relevant predictors (R^2^ = 0.37). This suggests increased motor-cognitive and working memory demands due to the additional multiple object tracking component, consistent with previous research in athletes highlighting the relevance of executive functioning in dual task agility performance [24]. However, the relatively high coefficient of variation (CV: ~ 31%) and wide confidence intervals, together with substantial interindividual variability (scores ranging from 5 to 184 points), suggest that the test may have been too challenging. A similar pattern was observed for the SKILLCOURT 2-back test, which exceeded the capabilities of approximately one quarter of participants (error rates > 30%). This indicates that both tasks may require further familiarization, adjusted difficulty levels (e.g., longer interstimulus intervals), or reconsideration of their suitability for older adults to ensure feasibility and internal validity. In addition, most participants were unable to complete the PC-based task switching test with an acceptable error rate, which was therefore not considered for analysis. Consequently, no corresponding PC-based reference was available for the SKILLCOURT task switching test, precluding the examination of its construct validity as well as that of the validity of the composite executive function score, which is based on all three subtests. Deviations in task setup and stimulus format between the SKILLCOURT and the corresponding PC-based versions (PsychoPy software) may have influenced cognitive processing, and motor response demands and may have affected the observed results.

### Practical implications

The findings have important practical implications. The SKILLCOURT demonstrates overall good reliability and sensitivity to performance changes across most agility and motor-cognitive assessments, making it suitable for performance evaluation and monitoring over time, although its practical application may be somewhat limited by cost and space requirements. Current standard assessments such as walking speed or the TUG test may not adequately capture the integrated cognitive demands of real-world mobility. In contrast, integrated motor–cognitive assessments such as the SKILLCOURT test battery may enhance ecological validity, making them a valuable complement to established motor- and cognitive-only tests, as they may better reflect motor–cognitive functions relevant to daily activities, falls, and cognitive decline [34]. Initial evidence supports this perspective, suggesting that motor-cognitive tasks such as the Stepping-TMT, stepping responses to inhibitory control stimuli, or the Trail-Walking-Test may be more sensitive to functional changes and more predictive of cognitive decline and falls in community-dwelling older adults than established motor measures (e.g., gait speed, TUG, functional reach, single-leg balance during single- and dual task conditions) or seated cognitive tests [30–32,76]. However, further research is required to establish reference values and to evaluate the added discriminative and predictive value of agility and motor–cognitive stepping assessments for health-related outcomes, such as falls and cognitive decline.

Our findings also highlight the importance of sufficient familiarization (e.g., separate session) and practice trials prior to formal testing to minimize learning effects and ensure that outcomes reflect true performance with high reproducibility. For cognitively more demanding motor-cognitive tasks, such as the executive function tasks, we recommend using response time measures that incorporate accuracy (e.g., the inverse efficiency score, IES) to account for speed-accuracy trade-offs. Additionally, composite scores integrating motor-cognitive performance across several subtests (e.g., SKILLCOURT composite executive function score) may provide a more robust and reliable estimate than single task outcomes. Not all SKILLCOURT tests may be suitable for older adults. In their current form, the 2-back and dual task agility tests overchallenged some participants, raising concerns about both feasibility and internal validity. Therefore, these tests cannot be recommended for this population in their present form.

## Conclusions

The results indicate generally good intersession reliability of most SKILLCOURT assessments for evaluating agility and motor-cognitive reactive stepping performance in community-dwelling, functionally independent older adults, with sensitivity to performance changes demonstrated across most test day comparisons. However, adequate familiarization is essential to ensure stable results. Construct validity was largely confirmed for the Random Star Run agility test. In contrast, the lower correlations observed for most motor-cognitive stepping tests compared with corresponding seated PC-based cognitive measures suggest that the addition of a more complex motor component may capture a distinct performance construct, potentially due to motor-cognitive interference. Future studies should further examine whether agility and motor-cognitive reactive stepping assessments provide added diagnostic value beyond established motor (single- and dual task) and seated cognitive assessments in distinguishing fallers from non-fallers and in predicting future fall risk or cognitive decline.

## Supporting information

S1 File Data file. (XLSX)
