# Supplementary material for: Reliability, construct validity, and usefulness of an assessment device for agility and motor-cognitive reactive stepping performance in community-dwelling older adults
Source: PLoS One. 2026 Jul 17;21(7):e0351843. doi: 10.1371/journal.pone.0351843 (PMC13379004; doi:10.1371/journal.pone.0351843)
Supplement: Attachment — Submitted filename: Response to Reviewers_R1.pdf [file pone.0351843.s003.pdf]

Dear Editor, dear Reviewers,

We would like to thank you for your thorough and constructive review, which has substantially improved the quality of the manuscript. Please find our responses below. We hope that the revised manuscript satisfactorily addresses your comments and concerns. All changes in the manuscript are highlighted in colored font.

Reviewer #1:

This is an extremely well-written manuscript. There is a thorough analysis and discussion, assessing different aspects of reliability and validity to paint a well-rounded view of this assessment battery. I had a few minor comments to address:

Response: We thank the reviewer for the thorough review. Please find our responses to your comments below. We greatly appreciate the positive feedback.

- Line 84-87: I had to read this sentence multiple times, and it was hard to make sense of it. Consider rephrasing and breaking it up into two sentences.

Response: We have rephrased this section accordingly.

- Line 94: change “stronger” to just “strong,” because it doesn’t appear you are making any comparisons. If you are trying to state that motor-cognitive tests have stronger associations than something else (such as single-task tests), please make that clarification.

Response: We have clarified this accordingly.

- Line 132-133: Please elaborate what you mean by “they familiarized themselves until they felt confident performing them?” Was this just by receiving instruction or did they practice the tests. If they practiced about how many times did they run through this. With the learning effects seen, this may provide some good context/clarity. You could also opt to address this in the discussion.

Response: During the first appointment, all SKILLCOURT tests were explained and demonstrated. Participants were given the opportunity to ask questions and completed at least one full familiarisation trial for each task. Additional practice trials were allowed until participants indicated that they understood the task. Familiarization procedures (instruction, demonstration, and practice trials) were standardised across all SKILLCOURT tests. We have added this information to the Methods section.

- Line 190-201: You state that they performed “reactive sidesteps,” but please clarify if they just had to complete one step or multiple. Additionally, was the sidestep(s) reaction time just the initiation of the movement? So, the time from the shape presentation to the first detected movement?

Response: Participants responded to a series of visual stimuli. Therefore, each test consisted of consecutive trials. We have clarified this accordingly. Regarding your second question, sidestep reaction time did not reflect movement initiation only. Instead, it was defined as the time from stimulus onset to the moment the participant reached the target square with the sidestep. Thus, the measure includes both reaction and movement time components. After reaching the target, participants immediately returned to the center position in preparation for the next trial. We have therefore changed the term to “response time” accordingly.

- Figure 3C: I would consider indicating that “ROT” is “red” in English. It took me a while to understand how the word “rot” would apply to the task until I remembered that it may be in German, then I had to look it up.

Response: We have corrected this accordingly.

- Line 296: You italicize the test name for all of the other tests; for consistency, please do that to the task switching test.

Response: We have now italicized all test names consistently throughout the manuscript.

- Line 408-414: The formatting of the post-hoc comparisons is hard to follow. Some are on a single line (like line 409) and sone have multiple to a line (like line 410). Please correct this and consider just making it a table.

Response: We have reorganized this information and summarized it in a separate table, as suggested.

- All figures are blurry, especially 1, 2, 5, and 6. Please provide better quality images.
Response: All figures have been updated and uploaded in 300 dpi resolution.

Reviewer #2:
PONE-D-26-10816
Journal: PLOS One
Date: 2026-04-23

Title: Reliability, Usefulness, and Construct Validity of an Instrumented System for Agility and Integrated Motor-Cognitive Assessment in functionally healthy Older Adults

This manuscript investigates cognitive-motor performance in older adults using SKILLCOURT-based assessments, including lower-extremity tasks targeting executive functions and agility test (Random Star Run) performed with and without an additional visual-cognitive load. Complementary measures of executive functions in a seated condition, as well as physical performance (e.g., TUG and walking speed) under single- and dual-task paradigm, were also evaluated, and their correlations were analyzed. In addition, the study examines reliability and practical utility.
Overall, the manuscript is well designed, clearly written, and presents comprehensive and interesting findings. Nevertheless, several aspects could be further improved to strengthen the work.

Response: We thank the reviewer for the thorough review. Please find our responses to your comments below. We greatly appreciate the positive feedback.

Major:
• Please clarify rationale for the statement that “lower correlations with standard cognitive tests are expected due to motor-cognitive interference” or specify it more precisely. While dual-task paradigms are introduced earlier, the underlying rationale for this hypothesis remains insufficiently explained, the effects of dual-task paradigms on cognitive and physical performance in older adults vary depending on task characteristics, such as postural control demands (e.g., Boisgontier et al., 2013; 10.1016/j.neubiorev.2013.07.014).

Response: Thank you for this helpful suggestion. We have revised this section and incorporated the concept of structural interference according to Boisgontier et al. (2013) in lines 67f. to better explain the rationale underlying our hypothesis. Based on this theoretical framework, we also revised the hypothesis in lines 102f. to clarify why lower correlations with standard cognitive tests may be expected in motor-cognitive dual-task settings.

• Participant characteristics are presented without stratification by age. However, the reported minimum values for MoCA and handgrip strength suggest that some individuals may not functionally healthy. The authors may therefore with to consider a more cautious characterization of the sample (e.g., “.. in functionally healthy older adults”, as used in the Discussion).
Response: Participant characteristics were presented for the total sample without age stratification, as age was included as a covariate in all main analyses, and stratified estimates of reliability and validity

were not part of the study aim. We agree that the reported MoCA and handgrip strength values suggest that some participants may not have been fully functionally healthy. Therefore, we have revised the characterization of the sample throughout the manuscript, including the title, to describe participants more cautiously as "functionally independent and community-dwelling" rather than "functionally healthy."

• The agility test on the SKILLCOURT, which primarily reflects physical functions, demonstrated good construct validity, whereas cognitive-motor tests on SKILLCOURT did not. Given the emphasis on cognitive-motor conditions in terms of ecological validity, further discussion and interpretation of these findings are encouraged (e.g., Kwag et al., 2026; 10.1038/s41598-026-44189-x)

Response: Thank you for making us aware of this interesting study. We have incorporated this reference into both the Introduction and the Discussion (practical implications) to further emphasize the potentially higher ecological validity of cognitive-motor assessments, despite their lower construct validity with standard cognitive and physical function tests. We also expanded the discussion of these findings accordingly (line 722f).

• The authors emphasize that the composite score of executive functions within the motor-cognitive tasks demonstrates superior performance, as reflected by higher ICCs across days and greater sensitivity compared to the individual components included in the motor-cognitive test (i.e., SKILLCOURT Test). However, the Stroop SKILLCOURT test appears to show comparable, and in some cases even higher, ICCs (e.g., between days 1-2) relative to the composite score. Moreover, the SKILLCOURT Stroop test demonstrates the best model fit among the motor-cognitive SKILLCOURT measures. It is also noteworthy that performance on traditional executive functions tests (in a seated condition) shows moderately stronger impact on predicting performance on SKILLCOURT Stroop performance than age. It may be helpful for the authors to elaborate on this aspect, as it could further strengthen the interpretation.

Response: The SKILLCOURT composite executive function score was calculated as the average performance across the three subtests (Stroop word-colour, task switching, and 2-back) and therefore does not represent a separate test. We have clarified this throughout the manuscript. We agree that the SKILLCOURT Stroop word-colour test was best explained by the corresponding PC-based Stroop test version, and we have highlighted this in the revised Discussion section (line 644f). Nevertheless, the R^2 value of 0.43 still suggests that greater motor demands and motor-cognitive interference, compared with PC-based assessments, may have influenced these relationships, although to a lesser extent than observed for the other motor-cognitive tests. Additionally, all validity analyses were adjusted for age.

Minor:

• The authors are encouraged to use terminology consistently when referring to the Stroop word-colour interference test. The version incorporated into the physical performance (using lower extremities) is described as reflecting 'interference control', whereas the seated version is described as reflecting 'conflict inhibition'. A more consistent terminology would improve clarity.

Response: Thank you for this suggestion. To improve consistency and clarity, we have standardized the terminology throughout the manuscript and now consistently refer to the test as the "Stroop word-colour" test, describing it as a measure of interference control.

• The purity of the Stroop word-colour interference test in the seated condition may warrant further clarification, as this task appears to involve not only inhibitory control but also elements of working memory (i.e., participants must remember the correspondence between colour and keyboard response). If a validation study exists supporting this interpretation, please provide a reference.

Response: In our paradigm, the stimulus–response mapping was continuously displayed (either on the screen in the motor–cognitive condition or via colour labels on the keyboard), such that participants were not required to memorize response mappings. Therefore, working memory demands were minimized. We have clarified this accordingly for both test versions.

• It is unclear why only the cognitive flexibility test in the seated condition differs in task format from the version incorporated into physical performance. In addition, the PC-based cognitive flexibility measure appears to be missing from Table 1.

Response: The PC-based task-switching version was too challenging for the present sample, with most participants unable to complete it within an acceptable error rate, and in several cases discontinuing the task. It was therefore removed from the analysis. We have clarified this in line 355f. Consequently, no corresponding PC-based reference was available for the SKILLCOURT task-switching test, precluding the examination of its construct validity, as well as the validity of the composite executive function score, which is based on all three subtests. This is also the reason why these test scores are not included in Table 1. These deviations in task design and stimulus format between the SKILLCOURT and the corresponding PC-based versions (PsychoPy software: task switching and 2-back) may have influenced cognitive processing and motor response demands and may have affected the observed results. We have added this to the Limitations section (line 715f).

• Table 2. The term "Number history of falls .." is unclear. It may be better to specify this as for example "number of individuals who experienced a fall …" or a similar clear formulation.

Response: We have adjusted this accordingly.

• No cognitive exclusion criterion was applied except for subjectively reported acute or chronic conditions, despite the cognitive sensitivity of the applied tests (particularly executive functions measures). It might therefore be informative to additionally examine whether results differ in participants with lower MoCA scores (e.g., < 22), which may indicate mild cognitive impairments.

Response: Thank you for this valuable comment. As no cognitive exclusion criteria were applied, cognitive function (MoCA) and age were tested as covariates but did not influence the main effects of the LMM (performance changes across test days) and were therefore not retained in the final models. Sensitivity analyses excluding participants with MoCA < 26 (indicative of potential cognitive impairment) yielded comparable results. We have added this to the Statistics section (line 385). The same applied to the construct validity analyses (line 415). This may be attributable to the repeated-measures (within-subject) test–retest design and the application of performance-based exclusion criteria, whereby test values with error rates exceeding 30% were excluded to minimize the influence of poor cognitive or motor performance and to preserve internal validity. Nevertheless, the sample cannot be considered entirely cognitively healthy, but rather functionally independent. We have added this to the Discussion (line 672f).

• Please clarify whether predictors were selectively entered into the regression models (enter model). If so, the rationale should be specified; for example, it is unclear why CRT-PC was included in both Random Star Rum and Dual-task agility models (see table 6A).

Response: We have added a section in the Methods clarifying the rationale for outcome selection (line 339f). The PC-based CRT was included in both the Random Star Run and Dual-task agility models, as both tasks require agility-related demands involving processing speed and response selection (i.e., selecting which target field to approach), based on previous evidence. However, we acknowledge that both tasks may rather reflect simple reaction time, as only one target field was presented, thereby requiring limited response selection. In older adults, who may be less familiar with such tasks, additional spatial translation processes (e.g., left/right/front/back mapping to the screen and court) may still be involved. Nevertheless, CRT did not remain a significant predictor for either test.

• Please improve the quality of Fig 6 to enhance clarity and readability.

Response: All figures have been updated and uploaded in 300 dpi resolution.

• Readability would be improved if task names are consistently and clearly distinguished throughout

the manuscript as either SKILLCOURT-based or tradition (seated) conditions, particularly in the Results section.

Response: We have adjusted the task names accordingly to improve consistency and clarity throughout the manuscript.
