# Supplementary material for: Reliability, construct validity, and usefulness of an assessment device for agility and motor-cognitive reactive stepping performance in community-dwelling older adults
Source: PLoS One. 2026 Jul 17;21(7):e0351843. doi: 10.1371/journal.pone.0351843 (PMC13379004; doi:10.1371/journal.pone.0351843)
Supplement: Peer review history

# Peer review: Decision Letter 0

**DOI:** 10.1371/journal.pone.0351843.r001

**Authors:** Filippo Bertozzi

24 Apr 2026

PONE-D-26-10816

Reliability, Usefulness, and Construct Validity of an Instrumented System for Agility and Integrated Motor-Cognitive Assessment in functionally healthy Older Adults

PLOS One

Dear Dr. Giesche,

Thank you for submitting your manuscript to PLOS ONE. After careful consideration, we feel that it has merit but does not fully meet PLOS ONE’s publication criteria as it currently stands. Therefore, we invite you to submit a revised version of the manuscript that addresses the points raised during the review process.

Please submit your revised manuscript by Jun 08 2026 11:59PM. If you will need more time than this to complete your revisions, please reply to this message or contact the journal office at plosone@plos.org. When you're ready to submit your revision, log on to https://www.editorialmanager.com/pone/ and select the 'Submissions Needing Revision' folder to locate your manuscript file.

Please include the following items when submitting your revised manuscript:

- A letter that responds to each point raised by the academic editor and reviewer(s). You should upload this letter as a separate file labeled 'Response to Reviewers'.
- A marked-up copy of your manuscript that highlights changes made to the original version. You should upload this as a separate file labeled 'Revised Manuscript with Track Changes'.
- An unmarked version of your revised paper without tracked changes. You should upload this as a separate file labeled 'Manuscript'.

If you would like to make changes to your financial disclosure, please include your updated statement in your cover letter. Guidelines for resubmitting your figure files are available below the reviewer comments at the end of this letter.

If applicable, we recommend that you deposit your laboratory protocols in protocols.io to enhance the reproducibility of your results. Protocols.io assigns your protocol its own identifier (DOI) so that it can be cited independently in the future. For instructions see: https://journals.plos.org/plosone/s/submission-guidelines#loc-laboratory-protocols. Additionally, PLOS ONE offers an option for publishing peer-reviewed Lab Protocol articles, which describe protocols hosted on protocols.io. Read more information on sharing protocols at https://plos.org/protocols?utm_medium=editorial-email&utm_source=authorletters&utm_campaign=protocols.

As the corresponding author, your ORCID iD is verified in the submission system and will appear in the published article. PLOS supports the use of ORCID, and we encourage all coauthors to register for an ORCID iD and use it as well. Please encourage your coauthors to verify their ORCID iD within the submission system before final acceptance, as unverified ORCID iDs will not appear in the published article. *Only*  the individual author can complete the verification step; PLOS staff *cannot*  verify ORCID iDs on behalf of authors.

We look forward to receiving your revised manuscript.

Kind regards,

Filippo Bertozzi, PhD

Academic Editor

PLOS One

Journal Requirements:

When submitting your revision, we need you to address these additional requirements.

1. Please ensure that your manuscript meets PLOS ONE's style requirements, including those for file naming. The PLOS ONE style templates can be found at

https://journals.plos.org/plosone/s/file?id=wjVg/PLOSOne_formatting_sample_main_body.pdf and

https://journals.plos.org/plosone/s/file?id=ba62/PLOSOne_formatting_sample_title_authors_affiliations.pdf

2. Thank you for stating the following in the Acknowledgments Section of your manuscript:

“The author(s) received no specific funding for this work.”

We note that you have provided funding information that is not currently declared in your Funding Statement. However, funding information should not appear in the Acknowledgments section or other areas of your manuscript. We will only publish funding information present in the Funding Statement section of the online submission form.

Please remove any funding-related text from the manuscript and let us know how you would like to update your Funding Statement. Currently, your Funding Statement reads as follows:

“The author(s) received no specific funding for this work.”

Please include your amended statements within your cover letter; we will change the online submission form on your behalf.

3. We note that Figures 2, 3 and 4 in your submission contain copyrighted images. All PLOS content is published under the Creative Commons Attribution License (CC BY 4.0), which means that the manuscript, images, and Supporting Information files will be freely available online, and any third party is permitted to access, download, copy, distribute, and use these materials in any way, even commercially, with proper attribution. For more information, see our copyright guidelines: http://journals.plos.org/plosone/s/licenses-and-copyright.

We require you to either (1) present written permission from the copyright holder to publish these figures specifically under the CC BY 4.0 license, or (2) remove the figures from your submission:

1. You may seek permission from the original copyright holder of Figure(s) [#] to publish the content specifically under the CC BY 4.0 license.

We recommend that you contact the original copyright holder with the Content Permission Form (http://journals.plos.org/plosone/s/file?id=7c09/content-permission-form.pdf) and the following text:

“I request permission for the open-access journal PLOS ONE to publish XXX under the Creative Commons Attribution License (CCAL) CC BY 4.0 (http://creativecommons.org/licenses/by/4.0/). Please be aware that this license allows unrestricted use and distribution, even commercially, by third parties. Please reply and provide explicit written permission to publish XXX under a CC BY license and complete the attached form.”

Please upload the completed Content Permission Form or other proof of granted permissions as an "Other" file with your submission.

In the figure caption of the copyrighted figure, please include the following text: “Reprinted from [ref] under a CC BY license, with permission from [name of publisher], original copyright [original copyright year].”

2. If you are unable to obtain permission from the original copyright holder to publish these figures under the CC BY 4.0 license or if the copyright holder’s requirements are incompatible with the CC BY 4.0 license, please either i) remove the figure or ii) supply a replacement figure that complies with the CC BY 4.0 license. Please check copyright information on all replacement figures and update the figure caption with source information. If applicable, please specify in the figure caption text when a figure is similar but not identical to the original image and is therefore for illustrative purposes only.

4. We note that there is identifying data in the Supporting Information file “Supporting information_data sheet.xlsx”. Due to the inclusion of these potentially identifying data, we have removed this file from your file inventory. Prior to sharing human research participant data, authors should consult with an ethics committee to ensure data are shared in accordance with participant consent and all applicable local laws.

Data sharing should never compromise participant privacy. It is therefore not appropriate to publicly share personally identifiable data on human research participants. The following are examples of data that should not be shared:

-Name, initials, physical address

-Ages more specific than whole numbers

-Internet protocol (IP) address

-Specific dates (birth dates, death dates, examination dates, etc.)

-Contact information such as phone number or email address

-Location data

-ID numbers that seem specific (long numbers, include initials, titled “Hospital ID”) rather than random (small numbers in numerical order)

Data that are not directly identifying may also be inappropriate to share, as in combination they can become identifying. For example, data collected from a small group of participants, vulnerable populations, or private groups should not be shared if they involve indirect identifiers (such as sex, ethnicity, location, etc.) that may risk the identification of study participants.

Additional guidance on preparing raw data for publication can be found in our Data Policy (https://journals.plos.org/plosone/s/data-availability#loc-human-research-participant-data-and-other-sensitive-data) and in the following article: http://www.bmj.com/content/340/bmj.c181.long.

Please remove or anonymize all personal information “ID numbers”, ensure that the data shared are in accordance with participant consent, and re-upload a fully anonymized data set. Please note that spreadsheet columns with personal information must be removed and not hidden as all hidden columns will appear in the published file.

5. If the reviewer comments include a recommendation to cite specific previously published works, please review and evaluate these publications to determine whether they are relevant and should be cited. There is no requirement to cite these works unless the editor has indicated otherwise.

[Note: HTML markup is below. Please do not edit.]

Reviewers' comments:

Reviewer's Responses to Questions

**Comments to the Author**

1. Is the manuscript technically sound, and do the data support the conclusions?

The manuscript must describe a technically sound piece of scientific research with data that supports the conclusions. Experiments must have been conducted rigorously, with appropriate controls, replication, and sample sizes. The conclusions must be drawn appropriately based on the data presented.

Reviewer #1: Yes

Reviewer #2: Yes

**********

2. Has the statistical analysis been performed appropriately and rigorously?

Reviewer #1: I Don't Know

Reviewer #2: Yes

**********

3. Have the authors made all data underlying the findings in their manuscript fully available?

The PLOS Data policy requires authors to make all data underlying the findings described in their manuscript fully available without restriction, with rare exception (please refer to the Data Availability Statement in the manuscript PDF file). The data should be provided as part of the manuscript or its supporting information, or deposited to a public repository. For example, in addition to summary statistics, the data points behind means, medians and variance measures should be available. If there are restrictions on publicly sharing data—e.g. participant privacy or use of data from a third party—those must be specified.

Reviewer #1: Yes

Reviewer #2: Yes

**********

4. Is the manuscript presented in an intelligible fashion and written in standard English?

PLOS ONE does not copyedit accepted manuscripts, so the language in submitted articles must be clear, correct, and unambiguous. Any typographical or grammatical errors should be corrected at revision, so please note any specific errors here.

Reviewer #1: Yes

Reviewer #2: Yes

**********

5. Review Comments to the Author

Please use the space provided to explain your answers to the questions above. You may also include additional comments for the author, including concerns about dual publication, research ethics, or publication ethics. (Please upload your review as an attachment if it exceeds 20,000 characters)

Reviewer #1: This is an extremely well-written manuscript. There is a thorough analysis and discussion, assessing different aspects of reliability and validity to paint a well-rounded view of this assessment battery. I had a few minor comments to address:

- Line 84-87: I had to read this sentence multiple times and it was hard to make sense of it. Consider rephrasing and breaking it up into two sentences.

- Line 94: change “stronger” to just “strong,” because it doesn’t appear you are making any comparisons. If you are trying to state that motor-cognitive tests have stronger associations than something else (such as single-task tests), please make that clarification.

- Line 132-133: Please elaborate what you mean by “they familiarized themselves until they felt confident performing them?” Was this just by receiving instruction or did they practice the tests. If they practiced about how many times did they run through this. With the learning effects seen, this may provide some good context/clarity. You could also opt to address this in the discussion.

- Line 190-201: You state that they performed “reactive sidesteps,” but please clarify if they just had to complete one step or multiple. Additionally, was the sidestep(s) reaction time just the initiation of the movement? So, the time from the shape presentation to the first detected movement?

- Figure 3C: I would consider indicating that “ROT” is “red” in English. It took me a while to understand how the word “rot” would apply to the task until I remembered that it may be in German, then I had to look it up.

- Line 296: You italicize the test name for all of the other tests; for consistency, please do that to the task switching test.

- Line 408-414: The formatting of the post-hoc comparisons is hard to follow. Some are on a single line (like line 409) and sone have multiple to a line (like line 410). Please correct this and consider just making it a table.

- All figures are blurry, especially 1, 2, 5, and 6. Please provide better quality images.

Reviewer #2: PONE-D-26-10816

Journal: PLOS One

Date: 2026-04-23

Title: Reliability, Usefulness, and Construct Validity of an Instrumented System for Agility and Integrated Motor-Cognitive Assessment in functionally healthy Older Adults

This manuscript investigates cognitive-motor performance in older adults using SKILLCOURT-based assessments, including lower-extremity tasks targeting executive functions and agility test (Random Star Run) performed with and without an additional visual-cognitive load. Complementary measures of executive functions in a seated condition, as well as physical performance (e.g., TUG and walking speed) under single- and dual-task paradigm, were also evaluated, and their correlations were analyzed. In addition, the study examines reliability and practical utility.

Overall, the manuscript is well designed, clearly written, and presents comprehensive and interesting findings. Nevertheless, several aspects could be further improved to strengthen the work.

Major:

• Please clarify rationale for the statement that “lower correlations with standard cognitive tests are expected due to motor-cognitive interference”, or specify it more precisely. While dual-task paradigms are introduced earlier, the underlying rationale for this hypothesis remains insufficiently explained, The effects of dual-task paradigms on cognitive and physical performance in older adults vary depending on task characteristics, such as postural control demands (e.g., Boisgontier et al., 2013; 10.1016/j.neubiorev.2013.07.014).

• Participant characteristics are presented without stratification by age. However, the reported minimum values for MoCA and handgrip strength suggest that some individuals may not functionally healthy. The authors may therefore with to consider a more cautious characterization of the sample (e.g., “.. in functionally healthy older adults”, as used in the Discussion).

• The agility test on the SKILLCOURT, which primarily reflects physical functions, demonstrated good construct validity, whereas cognitive-motor tests on SKILLCOURT did not. Given the emphasis on cognitive-motor conditions in terms of ecological validity, further discussion and interpretation of these findings are encouraged (e.g., Kwag et al., 2026; 10.1038/s41598-026-44189-x)

• The authors emphasize that the composite score of executive functions within the motor-cognitive tasks demonstrates superior performance, as reflected by higher ICCs across days and greater sensitivity compared to the individual components included in the motor-cognitive test (i.e., SKILLCOURT Test). However, the Stroop SKILLCOURT test appears to show comparable, and in some cases even higher, ICCs (e.g., between days 1-2) relative to the composite score. Moreover, the SKILLCOURT Stroop test demonstrates the best model fit among the motor-cognitive SKILLCOURT measures. It is also noteworthy that performance on traditional executive functions tests (in a seated condition) shows moderately stronger impact on predicting performance on SKILLCOURT Stroop performance than age. It may be helpful for the authors to elaborate on this aspect, as it could further strengthen the interpretation.

Minor:

• The authors are encouraged to use terminology consistently when referring to the Stroop word-colour intereference test. The version incorporated into the physical performance (using lower extremities) is described as reflecting ‘interference control’, whereas the seated version is described as reflecting ‘conflict inhibition’. A more consistent terminology would improve clarity.

• The purity of the Stroop word-colour interference test in the seated condition may warrant further clarification, as this task appears to involve not only inhibitory control but also elements of working memory (i.e., participants must remember the correspondence between colour and keyboard response). If a validation study exists supporting this interpretation, please provide a reference.

• It is unclear why only the cognitive flexibility test in the seated condition differs in task format from the version incorporated into physical performance. In addition, the PC-based cognitive flexibility measure appears to be missing from Table 1.

• Table 2. The term “Number history of falls ..” is unclear. It may be better to specify this as for example “number of individuals who experienced a fall …” or a similar clear formulation.

• No cognitive exclusion criterion was applied except for subjectively reported acute or chronic conditions, despite the cognitive sensitivity of the applied tests (particularly executive functions measures). It might therefore be informative to additionally examine whether results differ in participants with lower MoCA scores (e.g., < 22), which may indicate mild cognitive impairments.

• Please clarify whether predictors were selectively entered into the regression models (enter model). If so, the rationale should be specified; for example, it is unclear why CRT-PC was included in both Random Star Rum and Dual-task agility models (see table 6A).

• Please improve the quality of Fig 6 to enhance clarity and readability.

• Readability would be improved if task names are consistently and clearly distinguished throughout the manuscript as either SKILLCOURT-based or tradition (seated) conditions, particularly in the Results section.

**********

6. PLOS authors have the option to publish the peer review history of their article (what does this mean?). If published, this will include your full peer review and any attached files.

If you choose “no”, your identity will remain anonymous but your review may still be made public.

**Do you want your identity to be public for this peer review?**  For information about this choice, including consent withdrawal, please see our Privacy Policy.

Reviewer #1: No

Reviewer #2: No

**********

[NOTE: If reviewer comments were submitted as an attachment file, they will be attached to this email and accessible via the submission site. Please log into your account, locate the manuscript record, and check for the action link "View Attachments". If this link does not appear, there are no attachment files.]

To ensure your figures meet our technical requirements, please review our figure guidelines: https://journals.plos.org/plosone/s/figures

You may also use PLOS’s free figure tool, NAAS, to help you prepare publication quality figures: https://journals.plos.org/plosone/s/figures#loc-tools-for-figure-preparation.

NAAS will assess whether your figures meet our technical requirements by comparing each figure against our figure specifications.

# Author comment: Author response to Decision Letter 1

**DOI:** 10.1371/journal.pone.0351843.r002

21 May 2026

Dear Editor, dear Reviewers,

We would like to thank you for your thorough and constructive review, which has substantially improved the quality of the manuscript. Please find our responses below. We hope that the revised manuscript satisfactorily addresses your comments and concerns. All changes in the manuscript are highlighted in colored font.

Reviewer #1:

This is an extremely well-written manuscript. There is a thorough analysis and discussion, assessing different aspects of reliability and validity to paint a well-rounded view of this assessment battery. I had a few minor comments to address:

Response: We thank the reviewer for the thorough review. Please find our responses to your comments below. We greatly appreciate the positive feedback.

- Line 84-87: I had to read this sentence multiple times, and it was hard to make sense of it. Consider rephrasing and breaking it up into two sentences.

Response: We have rephrased this section accordingly.

- Line 94: change “stronger” to just “strong,” because it doesn’t appear you are making any comparisons. If you are trying to state that motor-cognitive tests have stronger associations than something else (such as single-task tests), please make that clarification.

Response: We have clarified this accordingly.

- Line 132-133: Please elaborate what you mean by “they familiarized themselves until they felt confident performing them?” Was this just by receiving instruction or did they practice the tests. If they practiced about how many times did they run through this. With the learning effects seen, this may provide some good context/clarity. You could also opt to address this in the discussion.

Response: During the first appointment, all SKILLCOURT tests were explained and demonstrated. Participants were given the opportunity to ask questions and completed at least one full familiarisation trial for each task. Additional practice trials were allowed until participants indicated that they understood the task. Familiarization procedures (instruction, demonstration, and practice trials) were standardised across all SKILLCOURT tests. We have added this information to the Methods section.

- Line 190-201: You state that they performed “reactive sidesteps,” but please clarify if they just had to complete one step or multiple. Additionally, was the sidestep(s) reaction time just the initiation of the movement? So, the time from the shape presentation to the first detected movement?

Response: Participants responded to a series of visual stimuli. Therefore, each test consisted of consecutive trials. We have clarified this accordingly. Regarding your second question, sidestep reaction time did not reflect movement initiation only. Instead, it was defined as the time from stimulus onset to the moment the participant reached the target square with the sidestep. Thus, the measure includes both reaction and movement time components. After reaching the target, participants immediately returned to the center position in preparation for the next trial. We have therefore changed the term to “response time” accordingly.

- Figure 3C: I would consider indicating that “ROT” is “red” in English. It took me a while to understand how the word “rot” would apply to the task until I remembered that it may be in German, then I had to look it up.

Response: We have corrected this accordingly.

- Line 296: You italicize the test name for all of the other tests; for consistency, please do that to the task switching test.

Response: We have now italicized all test names consistently throughout the manuscript.

- Line 408-414: The formatting of the post-hoc comparisons is hard to follow. Some are on a single line (like line 409) and sone have multiple to a line (like line 410). Please correct this and consider just making it a table.

Response: We have reorganized this information and summarized it in a separate table, as suggested.

- All figures are blurry, especially 1, 2, 5, and 6. Please provide better quality images.

Response: All figures have been updated and uploaded in 300 dpi resolution.

Reviewer #2:

PONE-D-26-10816

Journal: PLOS One

Date: 2026-04-23

Title: Reliability, Usefulness, and Construct Validity of an Instrumented System for Agility and Integrated Motor-Cognitive Assessment in functionally healthy Older Adults

This manuscript investigates cognitive-motor performance in older adults using SKILLCOURT-based assessments, including lower-extremity tasks targeting executive functions and agility test (Random Star Run) performed with and without an additional visual-cognitive load. Complementary measures of executive functions in a seated condition, as well as physical performance (e.g., TUG and walking speed) under single- and dual-task paradigm, were also evaluated, and their correlations were analyzed. In addition, the study examines reliability and practical utility.

Overall, the manuscript is well designed, clearly written, and presents comprehensive and interesting findings. Nevertheless, several aspects could be further improved to strengthen the work.

Response: We thank the reviewer for the thorough review. Please find our responses to your comments below. We greatly appreciate the positive feedback.

Major:

• Please clarify rationale for the statement that “lower correlations with standard cognitive tests are expected due to motor-cognitive interference” or specify it more precisely. While dual-task paradigms are introduced earlier, the underlying rationale for this hypothesis remains insufficiently explained, the effects of dual-task paradigms on cognitive and physical performance in older adults vary depending on task characteristics, such as postural control demands (e.g., Boisgontier et al., 2013; 10.1016/j.neubiorev.2013.07.014).

Response: Thank you for this helpful suggestion. We have revised this section and incorporated the concept of structural interference according to Boisgontier et al. (2013) in lines 67f. to better explain the rationale underlying our hypothesis. Based on this theoretical framework, we also revised the hypothesis in lines 102f. to clarify why lower correlations with standard cognitive tests may be expected in motor-cognitive dual-task settings.

• Participant characteristics are presented without stratification by age. However, the reported minimum values for MoCA and handgrip strength suggest that some individuals may not functionally healthy. The authors may therefore with to consider a more cautious characterization of the sample (e.g., “.. in functionally healthy older adults”, as used in the Discussion).

Response: Participant characteristics were presented for the total sample without age stratification, as age was included as a covariate in all main analyses, and stratified estimates of reliability and validity were not part of the study aim. We agree that the reported MoCA and handgrip strength values suggest that some participants may not have been fully functionally healthy. Therefore, we have revised the characterization of the sample throughout the manuscript, including the title, to describe participants more cautiously as “functionally independent and community-dwelling” rather than “functionally healthy.”

• The agility test on the SKILLCOURT, which primarily reflects physical functions, demonstrated good construct validity, whereas cognitive-motor tests on SKILLCOURT did not. Given the emphasis on cognitive-motor conditions in terms of ecological validity, further discussion and interpretation of these findings are encouraged (e.g., Kwag et al., 2026; 10.1038/s41598-026-44189-x)

Response: Thank you for making us aware of this interesting study. We have incorporated this reference into both the Introduction and the Discussion (practical implications) to further emphasize the potentially higher ecological validity of cognitive-motor assessments, despite their lower construct validity with standard cognitive and physical function tests. We also expanded the discussion of these findings accordingly (line 722f).

• The authors emphasize that the composite score of executive functions within the motor-cognitive tasks demonstrates superior performance, as reflected by higher ICCs across days and greater sensitivity compared to the individual components included in the motor-cognitive test (i.e., SKILLCOURT Test). However, the Stroop SKILLCOURT test appears to show comparable, and in some cases even higher, ICCs (e.g., between days 1-2) relative to the composite score. Moreover, the SKILLCOURT Stroop test demonstrates the best model fit among the motor-cognitive SKILLCOURT measures. It is also noteworthy that performance on traditional executive functions tests (in a seated condition) shows moderately stronger impact on predicting performance on SKILLCOURT Stroop performance than age. It may be helpful for the authors to elaborate on this aspect, as it could further strengthen the interpretation.

Response: The SKILLCOURT composite executive function score was calculated as the average performance across the three subtests (Stroop word-colour, task switching, and 2-back) and therefore does not represent a separate test. We have clarified this throughout the manuscript. We agree that the SKILLCOURT Stroop word-colour test was best explained by the corresponding PC-based Stroop test version, and we have highlighted this in the revised Discussion section (line 644f). Nevertheless, the R² value of 0.43 still suggests that greater motor demands and motor-cognitive interference, compared with PC-based assessments, may have influenced these relationships, although to a lesser extent than observed for the other motor-cognitive tests. Additionally, all validity analyses were adjusted for age.

Minor:

• The authors are encouraged to use terminology consistently when referring to the Stroop word-colour interference test. The version incorporated into the physical performance (using lower extremities) is described as reflecting ‘interference control’, whereas the seated version is described as reflecting ‘conflict inhibition’. A more consistent terminology would improve clarity.

Response: Thank you for this suggestion. To improve consistency and clarity, we have standardized the terminology throughout the manuscript and now consistently refer to the test as the “Stroop word-colour” test, describing it as a measure of interference control.

• The purity of the Stroop word-colour interference test in the seated condition may warrant further clarification, as this task appears to involve not only inhibitory control but also elements of working memory (i.e., participants must remember the correspondence between colour and keyboard response). If a validation study exists supporting this interpretation, please provide a reference.

Response: In our paradigm, the stimulus–response mapping was continuously displayed (either on the screen in the motor–cognitive condition or via colour labels on the keyboard), such that participants were not required to memorize response mappings. Therefore, working memory demands were minimized. We have clarified this accordingly for both test versions.

• It is unclear why only the cognitive flexibility test in the seated condition differs in task format from the version incorporated into physical performance. In addition, the PC-based cognitive flexibility measure appears to be missing from Table 1.

Response: The PC-based task-switching version was too challenging for the present sample, with most participants unable to complete it within an acceptable error rate, and in several cases discontinuing the task. It was therefore removed from the analysis. We have clarified this in line 355f. Consequently, no corresponding PC-based reference was available for the SKILLCOURT task-switching test, precluding the examination of its construct validity, as well as the validity of the composite executive function score, which is based on all three subtests. This is also the reason why these test scores are not included in Table 1. These deviations in task design and stimulus format between the SKILLCOURT and the corresponding PC-based versions (PsychoPy software: task switching and 2-back) may have influenced cognitive processing and motor response demands and may have affected the observed results. We have added this to the Limitations section (line 715f).

• Table 2. The term “Number history of falls ..” is unclear. It may be better to specify this as for example “number of individuals who experienced a fall …” or a similar clear formulation.

Response: We have adjusted this accordingly.

• No cognitive exclusion criterion was applied except for subjectively reported acute or chronic conditions, despite the cognitive sensitivity of the applied tests (particularly executive functions measures). It might therefore be informative to additionally examine whether results differ in participants with lower MoCA scores (e.g., < 22), which may indicate mild cognitive impairments.

Response: Thank you for this valuable comment. As no cognitive exclusion criteria were applied,

cognitive function (MoCA) and age were tested as covariates but did not influence the main effects of the LMM (performance changes across test days) and were therefore not retained in the final models. Sensitivity analyses excluding participants with MoCA < 26 (indicative of potential cognitive impairment) yielded comparable results. We have added this to the Statistics section (line 385). The same applied to the construct validity analyses (line 415). This may be attributable to the repeated-measures (within-subject) test–retest design and the application of performance-based exclusion criteria, whereby test values with error rates exceeding 30% were excluded to minimize the influence of poor cognitive or motor performance and to preserve internal validity. Nevertheless, the sample cannot be considered entirely cognitively healthy, but rather functionally independent. We have added this to the Discussion (line 672f).

• Please clarify whether predictors were selectively entered into the regression models (enter model). If so, the rationale should be specified; for example, it is unclear why CRT-PC was included in both Random Star Rum and Dual-task agility models (see table 6A).

Response: We have added a section in the Methods clarifying the rationale for outcome selection (line 339f). The PC-based CRT was included in both the Random Star Run and Dual-task agility models, as both tasks require agility-related demands involving processing speed and response selection (i.e., selecting which target field to approach), based on previous evidence. However, we acknowledge that both tasks may rather reflect simple reaction time, as only one target field was presented, thereby requiring limited response selection. In older adults, who may be less familiar with such tasks, additional spatial translation processes (e.g., left/right/front/back mapping to the screen and court) may still be involved. Nevertheless, CRT did not remain a significant predictor for either test.

• Please improve the quality of Fig 6 to enhance clarity and readability.

Response: All figures have been updated and uploaded in 300 dpi resolution.

• Readability would be improved if task names are consistently and clearly distinguished throughout the manuscript as either SKILLCOURT-based or tradition (seated) conditions, particularly in the Results section.

Response: We have adjusted the task names accordingly to improve consistency and clarity throughout the manuscript.

Attachment Submitted filename: Response to Reviewers_R1.pdf

# Peer review: Decision Letter 1

**DOI:** 10.1371/journal.pone.0351843.r003

**Authors:** Filippo Bertozzi, Filippo Bertozzi

3 Jun 2026

<p>Reliability, Construct Validity, and Usefulness of an Assessment Device for Agility and Motor-Cognitive Reactive Stepping Performance in Community-dwelling Older Adults

PONE-D-26-10816R1

Dear Dr. Giesche,

We’re pleased to inform you that your manuscript has been judged scientifically suitable for publication and will be formally accepted for publication once it meets all outstanding technical requirements.

Within one week, you’ll receive an e-mail detailing the required amendments. When these have been addressed, you’ll receive a formal acceptance letter and your manuscript will be scheduled for publication.

An invoice will be generated when your article is formally accepted. Please note, if your institution has a publishing partnership with PLOS and your article meets the relevant criteria, all or part of your publication costs will be covered. Please make sure your user information is up-to-date by logging into Editorial Manager at Editorial Manager® and clicking the ‘Update My Information' link at the top of the page. For questions related to billing, please contact billing support.

If your institution or institutions have a press office, please notify them about your upcoming paper to help maximize its impact. If they’ll be preparing press materials, please inform our press team as soon as possible -- no later than 48 hours after receiving the formal acceptance. Your manuscript will remain under strict press embargo until 2 pm Eastern Time on the date of publication. For more information, please contact onepress@plos.org.

Kind regards,

Filippo Bertozzi, PhD

Academic Editor

PLOS One

Additional Editor Comments (optional):

Reviewers' comments:

Reviewer's Responses to Questions

**Comments to the Author**

1. If the authors have adequately addressed your comments raised in a previous round of review and you feel that this manuscript is now acceptable for publication, you may indicate that here to bypass the “Comments to the Author” section, enter your conflict of interest statement in the “Confidential to Editor” section, and submit your "Accept" recommendation.

Reviewer #1: All comments have been addressed

Reviewer #2: All comments have been addressed

**********

2. Is the manuscript technically sound, and do the data support the conclusions?

The manuscript must describe a technically sound piece of scientific research with data that supports the conclusions. Experiments must have been conducted rigorously, with appropriate controls, replication, and sample sizes. The conclusions must be drawn appropriately based on the data presented.

Reviewer #1: Yes

Reviewer #2: Yes

**********

3. Has the statistical analysis been performed appropriately and rigorously?

Reviewer #1: I Don't Know

Reviewer #2: Yes

**********

4. Have the authors made all data underlying the findings in their manuscript fully available?

The PLOS Data policy requires authors to make all data underlying the findings described in their manuscript fully available without restriction, with rare exception (please refer to the Data Availability Statement in the manuscript PDF file). The data should be provided as part of the manuscript or its supporting information, or deposited to a public repository. For example, in addition to summary statistics, the data points behind means, medians and variance measures should be available. If there are restrictions on publicly sharing data—e.g. participant privacy or use of data from a third party—those must be specified.

Reviewer #1: Yes

Reviewer #2: Yes

**********

5. Is the manuscript presented in an intelligible fashion and written in standard English?

PLOS ONE does not copyedit accepted manuscripts, so the language in submitted articles must be clear, correct, and unambiguous. Any typographical or grammatical errors should be corrected at revision, so please note any specific errors here.

Reviewer #1: Yes

Reviewer #2: Yes

**********

6. Review Comments to the Author

Please use the space provided to explain your answers to the questions above. You may also include additional comments for the author, including concerns about dual publication, research ethics, or publication ethics. (Please upload your review as an attachment if it exceeds 20,000 characters)

Reviewer #1: (No Response)

Reviewer #2: The authors have responded thoroughly to the reviewer’s comments, and the manuscript has been

substantially improved. I have no further comments.

I believe that the findings presented in this paper make a valuable contribution to the field and

further advance the current body of knowledge.

Thank you.

**********

7. PLOS authors have the option to publish the peer review history of their article (what does this mean?). If published, this will include your full peer review and any attached files.

If you choose “no”, your identity will remain anonymous but your review may still be made public.

**Do you want your identity to be public for this peer review?**  For information about this choice, including consent withdrawal, please see our Privacy Policy.

Reviewer #1: No

Reviewer #2: No

**********

# Editor report: Acceptance letter

**DOI:** 10.1371/journal.pone.0351843.r004

**Authors:** Filippo Bertozzi, Filippo Bertozzi

PONE-D-26-10816R1

PLOS One

Dear Dr. Giesche,

I'm pleased to inform you that your manuscript has been deemed suitable for publication in PLOS One. Congratulations! Your manuscript is now being handed over to our production team.

At this stage, our production department will prepare your paper for publication. This includes ensuring the following:

* All references, tables, and figures are properly cited

* All relevant supporting information is included in the manuscript submission,

* There are no issues that prevent the paper from being properly typeset

You will receive further instructions from the production team, including instructions on how to review your proof when it is ready. Please keep in mind that we are working through a large volume of accepted articles, so please give us a few days to review your paper and let you know the next and final steps.

Lastly, if your institution or institutions have a press office, please let them know about your upcoming paper now to help maximize its impact. If they'll be preparing press materials, please inform our press team within the next 48 hours. Your manuscript will remain under strict press embargo until 2 pm Eastern Time on the date of publication. For more information, please contact onepress@plos.org.

You will receive an invoice from PLOS for your publication fee after your manuscript has reached the completed accept phase. If you receive an email requesting payment before acceptance or for any other service, this may be a phishing scheme. Learn how to identify phishing emails and protect your accounts at https://explore.plos.org/phishing.

If we can help with anything else, please email us at customercare@plos.org.

Thank you for submitting your work to PLOS ONE and supporting open access.

Kind regards,

PLOS ONE Editorial Office Staff

on behalf of

Dr. Filippo Bertozzi

Academic Editor

PLOS One
